# Preoptic area influences sleep-related seizures in a genetic epilepsy mouse model

**DOI:** 10.1093/cercor/bhaf187

**Published:** 2025-07-22

**Authors:** Cobie Victoria Potesta, Madeleine Sandra Cargile, Andrea Yan, Sarah Xiong, Robert L Macdonald, Martin J Gallagher, Chengwen Zhou

**Affiliations:** Department of Neurology, Vanderbilt University Medical Center, 1211 Medical Center Drive, Nashville, TN 37232, United States; Speech, Language and Hearing Science Program, Auburn University, Auburn, AL 36849, United States; Carroll Senior High School, Southlake, TX 76092, United States; Adlai E. Stevenson High School, Lincolnshire, IL 60069, United States; Department of Neurology, Vanderbilt University Medical Center, 1211 Medical Center Drive, Nashville, TN 37232, United States; Department of Neurology, Vanderbilt University Medical Center, 1211 Medical Center Drive, Nashville, TN 37232, United States; Vanderbilt Brain Institute and Neuroscience Graduate Program, Vanderbilt University Medical Center, Nashville, TN 37232, United States; Department of Neurology, Vanderbilt University Medical Center, 1211 Medical Center Drive, Nashville, TN 37232, United States; Vanderbilt Brain Institute and Neuroscience Graduate Program, Vanderbilt University Medical Center, Nashville, TN 37232, United States

**Keywords:** *Gabrg2^q390x^*, genetic seizures, optogenetic, preoptic area, sleep

## Abstract

In patients with refractory epilepsy, states of sleep and wakefulness affect the expression of seizures. However, the mechanism by which subcortical sleep circuitry affects seizures is unknown. Here, using *Gabrg2^Q390X^* knock-in (KI) genetic epileptic mouse model, we found that during sleep, subcortical preoptic area (POA) neurons were active in het *Gabrg2^Q390X^* KI mice and their activity preceded or/and coincided with epileptic (poly)spike–wave discharges. Optogenetic manipulating the POA activity altered sleep/wake periods in wild-type (wt) and the het *Gabrg2^Q390X^* KI mice. Most importantly, short-period optogenetic activation of epileptic cortical neurons alone did not effectively trigger seizures in the het *Gabrg2^Q390X^* KI mice, while optogenetic activation of the POA nucleus slightly influenced spontaneous epileptic activity in the het *Gabrg2^Q390X^* KI mice. In contrast, coordinated optogenetic activation/suppression of the subcortical POA nucleus with the optogenetic activation of epileptic cortical neurons effectively enhanced or suppressed epileptic activity in the het *Gabrg2^Q390X^* KI mice, indicating that the subcortical POA activation exacerbates seizures in the het *Gabrg2^Q390X^* KI mice*.* In addition, suppression of the subcortical POA nucleus decreased myoclonic jerks in the *Gabrg2^Q390X^* KI mice. Overall, this study reveals a circuit-based mechanism of sleep-preferential seizures in one genetic epilepsy model with implications for refractory epilepsy.

## Introduction

Sleep and wakefulness are daily physiological events (Saper et al. 2001, Saper et al. 2005, Saper et al. 2010, Scammell et al. 2017), while sleep spindles underlie memory consolidation (Niethard et al. 2018, Ngo et al. 2020, Girardeau and Lopes-Dos-Santos 2021, Skelin, Zhang et al. 2021) in the brain. Under pathological conditions in patients with epilepsy with strokes, brain injury, and genetic mutations, sleep can be altered resulting in memory/cognitive deficits (Beenhakker and Huguenard 2009, Ahmed and Vijayan 2014, Chauvette et al. 2016, Gibbon et al. 2019, Bergmann et al. 2020, Buchanan et al. 2021, Dell et al. 2021, Liguori et al. 2021). Moreover, sleep disturbance is one of comorbid symptoms being associated with patients with genetic and refractory epilepsy (Ahmed and Vijayan 2014, Gibbon et al. 2019, Bergmann et al. 2020, Manokaran et al. 2020, Dell et al. 2021, Pavlova et al. 2021) such as *Dravet* syndrome (Papale et al. 2013, Steel et al. 2017, Schoonjans et al. 2019) and frontal lobe epilepsy (Beleza and Pinho 2011). Moreover, sleep and wake states modulate seizure incidence in patients with genetic and refractory epilepsy (Ahmed and Vijayan 2014, Bagshaw et al. 2014, Caraballo, Pasteris et al. 2015, Bergmann et al. 2020, Dell et al. 2021) and genetic epileptic animal models (Papale et al. 2013, Catron et al. 2023). Our genetic mouse model with het *Gabrg2^Q390X^* mutation (for *Dravet* syndrome) exhibits an increased incidence of seizures during nonrapid eye movement (NREM) sleep (Catron et al. 2023). Thalamocortical circuitry (Contreras and Steriade 1995, Kandel and Buzsaki 1997, Timofeev and Steriade 1998) has been most extensively studied in the generation of sleep spindles and epileptic (poly)spike–wave discharges (SWD/PSDs) in animal models (Contreras et al. 1996, Contreras et al. 1997, Halasz et al. 2002, Huguenard and McCormick 2007) and patients (McCormick and Contreras 2001, Beenhakker and Huguenard 2009). However, the thalamocortical circuitry itself does not cause sleep/wake alteration and does not determine sleep-modulated ictal activity in genetic models and patients with refractory epilepsy (Bonakis and Koutroumanidis 2009, Halasz et al. 2013, Ng and Pavlova 2013, Ahmed and Vijayan 2014, Verbeek et al. 2015, Gibbon et al. 2019, Rashed et al. 2019, Konduru et al. 2021, Liguori et al. 2021, Van Nuland et al. 2021, Catron et al. 2023, Liang, Liang et al. 2024), suggesting that other circuitry mechanisms may be involved.

Sleep is controlled by subcortical structures including preoptic area (extended ventrolateral nucleus [VLPO] and median nucleus [MnPO] [Gvilia et al. 2017], referred as POA together in this manuscript) and ventrolateral periaqueductal gray (PAG) (Saper et al. 2001, Saper et al. 2005, Lim et al. 2014, Scammell et al. 2017). These nuclei suppress wake-promoting hypocretin/orexin neurons by their GABAergic axon terminals and cause the brain to shift to NREM sleep state under sleep pressure (Lu et al. 2000, Saper et al. 2001, Saper et al. 2005, Lim et al. 2014). Meanwhile, during NREM sleep, cortical neurons within thalamocortical circuitry exhibit up/down-state alterations (Shu et al. 2003, Battaglia et al. 2004, Dehghani et al. 2016, Levenstein et al. 2017, Niethard et al. 2018, Tukker et al. 2020, Cary and Turrigiano 2021, Torao-Angosto et al. 2021, Besing et al. 2022). Our previous work indicates that sleep-related up-states in cortical neurons (in slow-wave oscillations during NREM sleep) potentiate both excitatory and inhibitory synaptic strength in neurons (in a balanced way) from wild-type (wt) mice, while sleep-related up-states only potentiate excitatory synaptic strength in cortical neurons from heterozygous(het) *Gabrg2^Q390X^* knock-in (KI) mice, one *Dravet* syndrome genetic mouse model with *Gabrg2^Q390X^* mutation (Kang et al. 2015, Zhang et al. 2021, Catron et al. 2023). Compatible with this mechanism, spontaneous epileptic activity in the het *Gabrg2^Q390X^* KI mice occurs preferentially during NREM sleep (Catron et al. 2023), not during the awake period, and suppresses sleep spindle generation for memory consolidation. In addition, one prior study showed that subcortical MnPO activity can influence absence seizures in Wistar Albino Glaxo/Rijswijk (WAG/Rij) rats (Suntsova et al. 2009). Thus, we hypothesize that the subcortical POA neurons use this synaptic potentiation mechanism to trigger or regulate cortical seizures in the het *Gabrg2^Q390X^* KI mice during sleep. Although midbrain PAG also involves in sleep regulation (Weber et al. 2018, Zhong et al. 2019), due to PAG contributes to multiple functions such as pain/fear/anxiety/vocalization/defend-flight (Behbehani 1995, Dahlberg et al. 2018, Marciszewski et al. 2018, Marciszewski et al. 2019, Silva and McNaughton 2019, Wright and McDannald 2019), we chose the POA nucleus for this work to investigate the influence of sleep state on seizures.

The genetic *Gabrg2^Q390X^* KI mouse model (with a GABAergic γ2 subunit mutation of *Dravet* syndrome patient [Steel et al. 2017]) has been shown to exhibit spontaneous epileptic activity and other sleep disorders (Kang et al. 2015, Catron et al. 2023). Using this genetic *Gabrg2^Q390X^* KI mouse model with epilepsy during sleep and optogenetic method (Zhang et al. 2021, Catron et al. 2023), we found that during sleep, POA neurons were active and coincident with epileptic activity in the het *Gabrg2^Q390X^* KI mice and POA multi-unit activity preceded or/and coincided with cortical epileptic activity in the het *Gabrg2^Q390X^* KI mice. Further, coordinated manipulation of the subcortical POA activity with short activation of epileptic cortical neurons could trigger or suppress epilepsy in the het *Gabrg2^Q390X^* KI mice, supporting an operational mechanism that the subcortical POA nucleus influences cortical epileptic activity in this genetic epilepsy model during sleep, leading to memory and cognitive deficits in patients with epilepsy.

## Methods

### Mouse surgery for epidural EEG/optic cannula/tungsten electrode implantation

With all procedures in accordance with guidelines set by the institutional animal care and use committee of Vanderbilt University Medical Center, homozygous cFos-tTA::TetO transgenic mice were created by crossing cFos-tTA mice (#018306 from The Jackson Laboratory) (Reijmers et al. 2007) and TetO mice (#017906, Tg(tetO-hop/EGFP,-COP4/mCherry)6Kftnk/J, from The Jackson Laboratory) (Chuhma et al. 2011). The homozygous mice then crossed with het *Gabrg2^Q390X^* KI mice to breed cFos-tTA::TetO::wt littermates and cFos-tTA::TetO::het *Gabrg2^Q390X^* KI mice (*shortened as wt and het mice unless specified*) to activity-dependently express both halorhodopsin (NpHR) and channelrhodopsin (ChR2) proteins in neurons within cerebral cortex and subcortical structures for the optogenetic manipulation of cortical/subcortical activity (no doxycycline was used for these mice due to that febrile seizures and generalized tonic–clonic seizures occur during both early development and adult period) (Reijmers et al. 2007, Kang et al. 2015, Warner et al. 2017). Particularly, compared to wt mice, cFos-tTA::TetO::het *Gabrg2^Q390X^* KI mice have spontaneous epileptic activity (Catron et al. 2023) during sleep to drive NpHR and ChR2 expression within epileptic neurons. In the cFos-tTA:tetO::wt and cFos-tTA::tetO::het *Gabrg2^Q390X^* KI mice, the NpHR protein expression in the brain was confirmed by checking green GFP expression in neurons and the ChR2 protein expression by red mCherry expression in neurons (further with antibody against mCherry) within brain slices or post-paraformaldehyde (PFA) fixed brain sections (see cell counting section). cFos-GFP mice (#014135, from The Jackson Laboratory) were also used to breed cFos-GFP::wt and cFos-GFP::*het Gabrg2^Q390X^* KI mice for tracking cFos-GFP positive neurons within the whole brain. After anesthetized with 1% to 3% isoflurane (vol/vol) during aseptic surgery, these wt and het mice (both genders, 2 to 5 mo old) were implanted with three epidural EEG screw electrodes (each for one hemisphere and one for grounding above cerebellum, Pinnacle Technology, #8201). Meanwhile, depending on experiments, two fiber optic cannulas (0.2 to 0.4 mm diameter, Thorlabs Inc., Newton, New Jersey) for laser light delivery in vivo were also implanted in somatosensory cortex and subcortical POA (extended VLPO and MnPO nuclei [Lu et al. 2000]) (brain atlas coordinates: somatosensory cortex range, anterior–posterior between −1.82 and −0.46 mm, midline-lateral between +2.0 and +4.0 mm reference to bregma, dorsal-ventral 0.7 to 1.1 mm reference to pia surface; POA, anterior–posterior between +0.4 and +0.2 mm, midline-lateral between 0.3 and 0.5 mm reference to bregma, dorsal-ventral 5.5 to 5.7 mm reference to pia surface, Supplemental Fig. S4 for optic cannula track within VLPO). When necessary, EEG screw electrode positions were adjusted for the space of optic cannula implantation. In addition, one bipolar tungsten electrode was implanted into the POA nucleus in some experiments. Tungsten electrode/optic cannula placements within the brain were checked after euthanasia. One pair of electromyography (EMG) leads were inserted into the trapezius muscles to monitor mouse motor activity such as head movements and staring along with simultaneous videos.

### E‌EG/multi-unit recordings and optogenetic manipulation in vivo

**Fig. 1:**
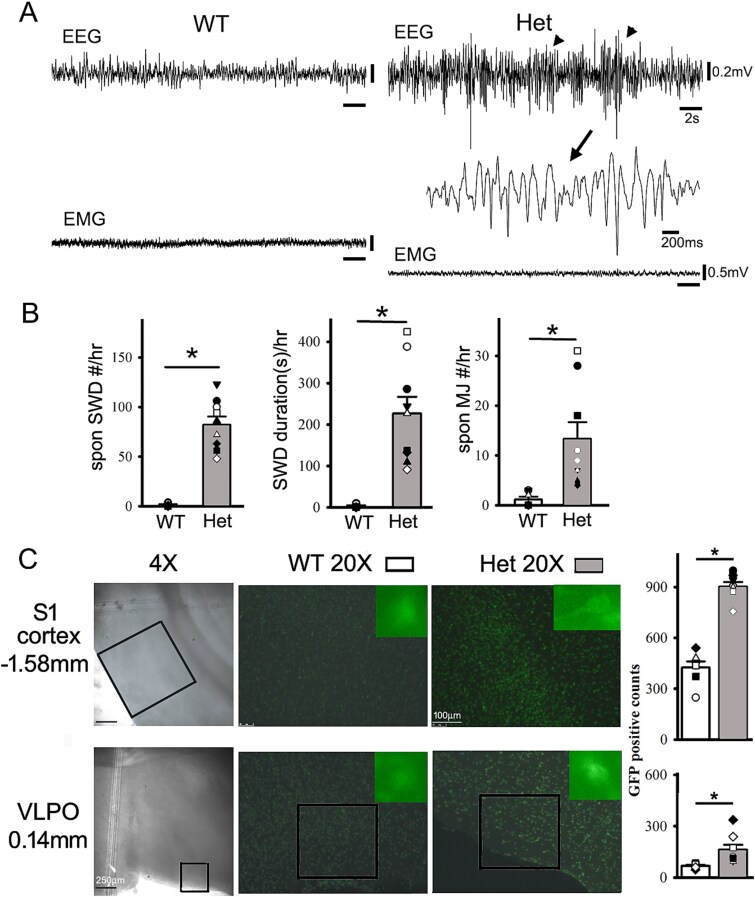
Compared to age-matched wt mice, more epileptic activity is present in het *Gabrg2^Q390X^* KI mice during sleep, with more cFos-GFP positive neurons within somatosensory cortex (S1) and POA (extended VLPO). Panel A shows simultaneous representative EEG (band filtered 0.1 to 100 Hz) and EMG recordings (band filtered 10 to 400 Hz) from wt and the het *Gabrg2^Q390X^* KI mice during NREM sleep, with panel B shows summary data of spontaneous epileptic activity and MJs in the mice. One SWD event is expanded in small temporal scales. The arrowheads in the EEG traces indicate the detected epileptic SWD/PSDs in the het *Gabrg2^Q390X^* KI mice during NREM sleep. Panel B shows summary data (*each data point represents original data from one single mouse*) with wt *n* = 7 mice, het *n* = 9 mice, t-test *P* = 0.0005 for SWD/hr, *P* = 0.0006 for SWD/PSD duration(s)#/hr, *P* = 0.007 for MJ#/hr. Left panels in panel C show 4x DIC images brain sections with counting boxes within the somatosensory cortex (S1 cortex) and the POA and landmarks (hippocampus for S1 cortex and ventral third ventricles/anterior commissure for extended VLPO nucleus [Lu et al. 2000]), with mouse brain atlas bregma coordinates in the far left. Middle and right panels show wt and het 20x section (with cFos-GFP positive puncta) of brain areas from the cFos-GFP::Wt and het cFos-GFP::*Gabrg2^Q390X^* KI mice (wt *n* = 6 and het *n* = 7 mice) (*each data point represents averaged counting values per section or counting box from the same mouse [averaged from three sections for both sides*]). Single GFP-positive neurons (at 40x) are shown as inserts in the 20x panels. The puncta within the whole somatosensory cortex section (20x) were counted and the counting boxes within 20x sections are shown for extended VLPO cFos-GFP puncta counting. Scale bars are indicated as labeled. * significant change with t-test *P* < 0.05.

As reported previously, (Catron et al. 2023), postsurgery mice were continuously monitored for recovery from anesthesia and remained in the animal care facility for 5 to 7 d (normal wake/sleep circadian rhythm) before simultaneous EEG/multi-unit activity recordings in vivo*,* along with epileptic behavior being video-recorded for Racine-stage scoring. Two-channel EEG (band pass [bp] filtered at 0.1 to 100 Hz) and one-channel POA multi-unit recordings (bp filtered 300 to 10 KHz) along with one channel EMG(bp filtered 10 to 400 Hz) were collected by using two multiClamp 700B amplifiers (total four channels and all in current-clamp mode) (Molecular devices Inc., Union City, CA) and Clampex 10 software (Molecular Devices Inc., Union City, CA), and digitized at 20 kHz using a Digidata 1440A. An optic fiber cable was connected to the optic cannula to deliver laser, which was controlled by a yellow DPSS laser for NpHR (MGL-III-589-50 [50 mW, Ultralazers Co., Inc]) or a blue DPSS laser for ChR2 (MBL-III-473-100 [100 mW, Ultralazers Co., Inc]). And the laser delivery timing was controlled by Clampex 10 software for optogenetic manipulation of POA and cortex. Each optogenetic manipulation cycle (2 s, see Figs 5B and E, 6B and E) consists of one POA manipulation (1,900 ms long) and one cortex manipulation (100 ms long). *The optogenetic manipulation was repeated 300 cycles (total 10 min)*. During the POA-manipulation (1,900 ms) of each cycle, blue laser was activated at 20 Hz (1 ms for each pulse duration) for POA-ChR2 activation (Abbott et al. 2009, Vesuna et al. 2020) or yellow laser was continuously activated for POA-NpHR suppression (Gradinaru et al. 2007, Vesuna et al. 2020). During the cortex-manipulation (100 ms long) of each cycle, blue laser was activated at 20 Hz for cortex (S1)-ChR2 activation (60 ms long) (simulating neuronal up-states for sleep spindles) (Kandel and Buzsaki 1997). The intensity was determined by slice experiments ex vivo (see brain slice section). For some experiments, POA-manipulation alone or cortex manipulation alone was conducted in mice. When combined cortex and POA optogenetic manipulations were used, the temporal sequence of POA-manipulation and cortex-manipulation were coordinated to simulate cortical neuronal up/down-states within slow-wave oscillations during NREM sleep (see Figs. 5B and E, 6B and E). Single mice could be conducted with both POA activation and suppression with at least 1 to 2 h intervals and without specific temporal sequels. Thus, precontrol SWD/PSD data in mice (except Fig. 1) were mixed from fresh start mouse precontrol data and postoptogenetic manipulation mice for summary data.

Mouse epileptic EEG activity was defined as bilateral synchronous (poly)spike–wave discharges (PSDs) (SWDs, 6-12 Hz) with duration > 1 s and voltage amplitudes at least 2-fold higher than baseline amplitude. During NREM sleep period, SWD/PSD amplitudes were set as at least 1.5X of baseline amplitude due to large delta EEG amplitude. All atypical and typical absence epilepsy and general tonic–clonic seizures (GTCSs) started with SWD/PSDs, accompanied by characteristic motor behaviors. The behavior associated with SWD/PSDs consisted of immobility, facial myoclonus, and vibrissal twitching. The SWD/PSDs and animal epileptic behaviors were also checked and confirmed by persons blind to animal genotypes. The onset times of SWD/PSDs were determined by their leading-edge points crossing (either upward or downward) the precedent EEG baseline. PSDs will be identified as very brief (< 0.5 s), high frequency (approximately 50 ms interspike interval) complexes (Garcia-Cabrero et al. 2012, Arain et al. 2015) with voltages at least twice the background voltage. SWDs have a lower spike frequency (interspike interval 125 to 166 ms), and a much higher rhythmicity than the PSDs (Arain et al. 2015). PSDs often coincide with myoclonic jerks and SWDs. Epileptic myoclonic jerks (Arain et al. 2015, Ding and Gallagher 2016, Ding et al. 2019) will be identified as very brief (<1 s), lightening-like body spasms associated with PSDs (Yu et al. 2006). Interictal spikes(IS) will be detected by at least 5x standard deviation (with durations < 100 ms) above baseline EEG mean amplitudes (Levesque et al. 2021, Levesque et al. 2023) to prevent false positive detection (Ding et al. 2019). *Unlike IS, myoclonic seizures (jerks) are associated with head/body/tail-limbs jerks* (Ding et al. 2019). Due to the short experiment time of optogenetic manipulation, GTCSs in the het *Gabrg2^Q390X^* KI mice were very rare and not examined in this study. High-frequency tungsten multi-unit activity was obtained by postexperiment band-filtering (400 ~ 800 Hz) originally recorded tungsten electrode activity band-filtered at 300-3 KHz in vivo. One threshold detection method (at least 2x baseline amplitude) was used to detect high-frequency activity events using Clampfit 10 software (Molecular Devices Inc., Molecular Devices Inc., Union City, CA) and their duration was analyzed. Any high-frequency activity associated with motor behaviors (video monitored) was removed from this analysis. *Each epilepsy, sleep/wake and sleep spindle data point in the result section and figures represented original data from one single mouse and data from all mice were processed for further group comparison and statistical analysis with mean ± SEM (standard error)*. The Bonferroni correction was used for multiple comparison correction when necessary.

Sleep period was determined as period without nuchal EMG activity (active sleep) or just twitch activity (quiet sleep), while wake period was defined as large amplitude EMG activity (Catron et al. 2023). Due to 10-min optogenetic manipulation, we converted pre-optogenetic absolute values of sleep/wake period for 1 h as the percentage (total as 100%). Then, postoptogenetic absolute values of sleep/wake period for 1 h (chosen by the temporal effect during 1 h of slow-wave oscillation (SWO)-induced synaptic sEPSC potentiation, Fig. 7H of [Zhang et al. 2021]) were converted to percentage changes of pre-optogenetic absolute sleep/wake values. The spike-sorting method was used to identify multi-unit activity within POA (including both VLPO and MnPO nuclei) during sleep period, by using the spike amplitudes and spike width (Mohns et al. 2006). These units were confirmed as sleep-selective POA activity and they were further examined whether they preceded and/or synchronized with SWD/PSDs. The intervals were defined as the time between the end of the POA multi-unit activity and the adjacent SWD/PSDs (shorten as POA-SWD/PSDs). If multiple SWD/PSDs followed the POA multi-unit activity, we used the first SWD/PSD for the POA-SWD/PSD intervals. In a similar way, if multiple POA multi-unit activities preceded the SWD/PSDs, only the last POA activity were used to determine the intervals. If the POA multi-unit activities synchronized with the SWD/PSDs, we would use the leading point of the POA multi-units and the leading points of the SWD/PSDs. Therefore, we focused on the temporal order/sequences between POA single/multi-unit activity and subsequent SWD/PSDs. *Several spike-sorted POA single-units in the result section and figures represented original data from one single mouse and data from all mice were processed for further group comparison and statistical analysis with mean ± SEM (standard error)*.

### cFos-positive puncta/cell counting

The mice used for histology studies were transcardially perfused with PFA (4% in phosphate buffered saline [PBS]) to fix brain tissues and the fixed mouse brains were sectioned in 70 μm thickness to examine cFos-GFP positive neurons within somatosensory cortex and subcortical preoptic area (which included the implantation area of optic cannulas) from both cFos-GFP::wt (or cFos-tTA:tetO::wt) and cFos-GFP::het *Gabrg2^Q390X^* KI mice (or cFos-tTA::tetO::het *Gabrg2^Q390X^* KI mice). Neurons were also stained with 4′,6-diamidino-2-phenylindole as DAPI (DAPI) (VECTASHIELD® mounting media H-1500, Vector Laboratories Inc. Burlingame CA) for nucleus staining. Under one fluorescence microscope (Leica dm6000B, Leica Microsystems Inc., Buffalo Grove, IL) or Zeiss LSM-710 inverted confocal microscope, the cFos-GFP positive neurons within the same brain sections were counted within a fixed size counting box. Within mouse brain atlas S1 cortex coordinates between −1.82 and −0.46 mm (anterior–posterior), three coronal sections (for each mouse, anterior–posterior) around the −1.5 mm (optic cannula implantation site) were used with 200 mm apart. Within mouse brain atlas POA coordinates between anterior–posterior +0.5 ~ +0.2 mm that cover the implantation coordinates of optic cannulas, the MnPO nucleus was framed by the top of third ventricle and the ventral side of medium septum (Suntsova et al. 2002) and the VLPO nucleus was framed by the lateral edge of third ventricle and extended 250 μm dorsallly and 300 ~ 800 μm laterally in three coronal sections (Lu et al. 2000). These neuron counts (*total within three sections/both sides*) were further analyzed with Image-J program (downloaded from NIH website), with different thresholding levels. *Each counting data point in the result section and figures represented one mean value averaged for all sections from the same one mouse and mean values from all mice were processed for further group comparison and statistical analysis with mean ± SEM (standard error)*.

### Brain slice preparation and recordings

Brain slice preparation has been described in our previous work (Zhang et al. 2021). Both wt littermates and het *Gabrg2^Q390X^* KI mice aged P60–150 (Kang et al. 2015) were used in this study. After mice were deeply anesthetized and decapitated, mouse brains were sectioned with a vibratome (Leica VT 1200S, Leica Biosystems Inc) for coronal brain slices (cortex 300 to 320 μm thickness, containing somatosensory cortex or 200 ~ 250 μm for VLPO/MnPO nuclei) in a sucrose-based ice-cold solution (containing [in mM]): 214 sucrose, 2.5 KCl, 1.25 NaH_2_PO_4_, 0.5 CaCl_2_, 10 MgSO_4_, 24 NaHCO_3_, and 11 D-glucose, pH 7.4) and brain slices were incubated in a chamber at 35°C to 36°C for 40 min with continuously oxygenated artificial cerebral spinal fluid (ACSF) (see below for components). Finally slices remained at room temperature for at least 1 h before electrophysiological recordings at 32°C. By using a Nikon infrared/DIC microscope (Eclipse FN1, Nikon Corp. Inc), whole-cell patch-clamp recordings (voltage- or current-clamp) were made from somatosensory cortical cFos-GFP positive layer V pyramidal neurons or VLPO/MnPO neurons while slices were continuously superfused (flow speed 1 to 1.5 mL/min) with ACSF (containing [mM]: 126 NaCl, 2.5 KCl, 1.25 NaH_2_PO_4_, 2 CaCl_2_, 2 MgCl_2_, 26 NaHCO_3_, 10 D-glucose, pH 7.4) bubbled with 95% O_2_/5% CO_2_. Using an internal solution (consisted of [in mM]: 120 K-gluconate, 11 KCl, 1 MgCl_2_, 1 CaCl_2_, 0.6 EGTA, 10 HEPES, 2 Na-ATP, 0.6 Na-GTP, 10 K-creatine-phosphate, pH 7.3 and filled electrodes with resistances of 2 ~ 5 MΩ), action potentials were recorded with varied current injection from resting membrane potentials. Access resistance Ra was continuously monitored and recordings with Ra larger than 25 MΩ or 20% change were discarded.

Data were collected by using one multiClamp 700B amplifier (filtered at 2 kHz) and digitized at 20 kHz by using one Digidata 1440A (Molecular Devices Inc., Union City, CA), along with Clampex 10 software (Molecular Devices Inc., Union City, CA). When necessary, *several cells from each slice and each mouse were recorded and all data were used for group average/comparison*. All figures were prepared with both Microsoft Excel, SigmaPlot, and Adobe Photoshop softwares. Data were expressed as mean ± SEM (standard error). Statistics were performed with student paired t-test or one/two-way ANOVA with *P* < 0.05 as significance when necessary. The Bonferroni correction was used for multiple comparison correction when necessary.

## Results

### Seizure or sleep-related cFos-GFP positive neurons are present in somatosensory cortex and POA (extended VLPO) in het *Gabrg2^Q390X^* KI mice

Sleep has been shown to modulate seizure activity in human patients, particularly for refractory epilepsy (Ahmed and Vijayan 2014, Bagshaw et al. 2014, Caraballo et al. 2015, Bergmann et al. 2020, Dell et al. 2021) and genetic epileptic animal models (Papale et al. 2013, Catron et al. 2023). Similar to be our previous work (Catron et al. 2023), compared to wt mice, het *Gabrg2^Q390X^* KI mice have spontaneous epileptic activity during sleep (Fig. 1A and B) (SWD/PSD #/hr: wt 1.428 ± 3.519, *n* = 7 mice and het 82.00 ± 8.38, *n* = 9 mice, t-test *P* = 0.0005; SWD/PSD duration[s]#/hr: wt 3.16 ± 1.53 and het 226.59 ± 40.3, t-test *P* = 0.0006; MJ#/hr: wt 1.14 ± 0.55 and het 13.33 ± 3.35, t-test *P* = 0.007). (The epileptic activity in the mice were acquired before any optogenetic manipulation of cortex and POA nucleus.) Thus, *spontaneous seizures in the mice were used to activity-dependently label epileptic neurons (*Barth et al. 2004*,*  Reijmers et al. 2007*) in the brain*. Then we crossed cFos-GFP transgenic mice with het *Gabrg2^Q390X^* KI mice to examine whether cFos-GFP positive neurons (Barth et al. 2004, Roy et al. 2022) were present within cortex and subcortical structures for epileptic network and sleep circuitry (Reijmers et al. 2007, Burnsed et al. 2019). With cFos::wt and cFos::*Gabrg2^Q390X^* KI mice, we identified the cFos-GFP positive neurons within the somatosensory cortex (epileptic neuron engram/ensembles) and the subcortical POA (sleep circuitry). Compared to the wt mice (*n* = 7 mice, cortex S1 wt 20X 425.28 ± 35.19/per section and *n* = 8, POA wt 20X 68.62 ± 4.84/per counting box), the het *Gabrg2^Q390X^* KI mice (*n* = 9 mice) exhibited more GFP-positive epileptic neurons within the somatosensory cortex (Fig. 1C, cortex S1 20X het GFP-positive puncta 901.14 ± 28.01/per section, t-test *P* < 0.001) and the subcortical POA (Fig. 1C, extended VLPO 20X het 161.75 ± 30.34/per counting box, *n* = 8, t-test *P* = 0.008), indicating that sleep-circuitry was more active (likely) and coincident with epileptic activity from the het *Gabrg2^Q390X^* mice, compatible with our recent work on the NREM sleep preference of epileptic activity in the genetic *Gabrg2^Q390X^* KI epilepsy model (Catron et al. 2023).

### POA neuronal multi-unit activity precedes epileptic discharges in het *Gabrg2^Q390X^* KI mice

**Fig. 2:**
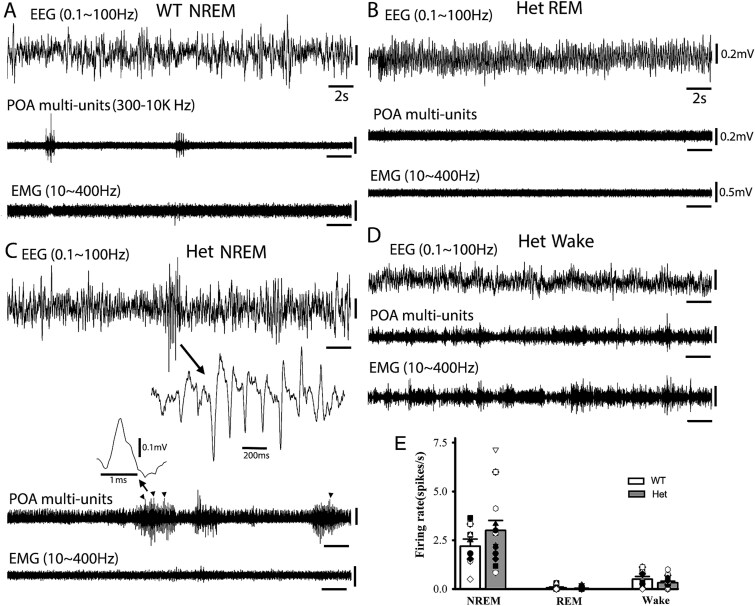
POA neuron firings precede or/and coincide with epileptic SWD/PSDs in het *Gabrg2^Q390X^* KI mice during NREM sleep. Panels A–D show simultaneous representative EEG (band filtered 0.1 to 100 Hz)/POA multi-unit tungsten electrode recordings (band filtered 300 to 10 KHz) and EMG recordings (band filtered 10 to 400 Hz) from wt and the het *Gabrg2^Q390X^* KI mice during NREM sleep, REM sleep and wake periods. In panel C, one SWD event is expanded in small temporal scale from EEG traces and one isolated single-unit before/upon SWD events is expanded in small temporal/amplitude scales for spike-sorting. The arrowheads in the POA multi-unit trace indicate the same detected single-unit firings from the het *Gabrg2^Q390X^* KI mice during NREM sleep. Scale bars are indicated as labeled. Panel E shows firing change of the same spike-sorted POA single-units across NREM/REM sleep and wake periods (*n* = 8 POA single-from wt and *n* = 13 POA single-units from het mice) (*n* = 4 wt and *n* = 5 het mice). *Each data point represents original spike-sorted POA neuronal firings from wt or het mice* and significant change with one-way ANOVA (for NREM vs REM vs wake, *P* < 0.001 for both wt and het mice).

Furthermore, we examined whether POA neuronal activity was real-time correlated with seizures in the het *Gabrg2^Q390X^* KI mice. Multi-unit recordings were used to record neuronal activity within the POA from mice and single-unit activity was isolated for spike-sorting (Suntsova et al. 2002) to identify the same neurons being active during NREM/REM sleep and seizures. In both the wt and het *Gabrg2^Q390X^* KI mice, most spike-sorted POA neurons were selectively active during NREM sleep (wt 2.19 ± 0.37 Hz, *n* = 8 from 4 mice; het 3.00 ± 0.509 Hz, *n* = 13 from 5 mice, not significant between wt and het group) and much less active during REM sleep (wt 0.06 ± 0.04 Hz; het 0.02 ± 0.02 Hz, not significant between wt and het group) and wake period (wt 0.50 ± 0.14 Hz; het 0.32 ± 0.08 Hz, not significant between wt and het group) (Fig. 2A and C) (One-way ANOVA for NREM vs REM vs wake, *P* < 0.001 for both wt and het mice), suggesting that these POA neurons were sleep-related and involved in sleep-state regulation (Lu et al. 2000, Suntsova et al. 2002, Alam et al. 2014). However, some POA neuronal firings (in very low-frequency) do not show sleep or wake modulation (wt *n* = 3, het *n* = 4).

In the het *Gabrg2^Q30X^* KI mice (not wt mice), some POA neurons were active and immediately preceded (627.09 ± 150.60 ms, *n* = 7 from 5 het mice) or/and coincided with ictal SWD/PSDs discharges (Fig. 2C arrowheads). These POA neurons were mostly/preferentially active during sleep/nonmotor state (not wake period), indicating that these POA neurons likely regulated sleep brain state for subsequent ictal discharges in the het *Gabrg2^Q390X^* KI mice.

### Sleep and arousal/awake states can be altered by sole optogenetic manipulation of subcortical POA nucleus in mice in vivo

To optogenetically manipulate subcortical l POA neurons during sleep state and upon seizures from het *Gabrg2^Q390X^* KI mice, we crossed the het *Gabrg2^Q390X^* KI mice with cFos-tTA::TetO mice (Reijmers et al. 2007) to express ChR2 and NpHR proteins in cFos-GFP/mCherry positive neurons within the cortex and subcortical POA from the wt and het *Gabrg2^Q390X^* KI mice (no doxycycline given). The cFos-GFP protein expression patterns in the het cFos-tTA::TetO::*Gabrg2^Q390X^* KI mice (Supplemental Fig. S1A het) were very similar to the those in the het cFos::*Gabrg2^Q390X^* mice (Fig. 1C, cFos-GFP puncta in S1-cortex). By delivering blue laser light at 20 Hz to activate ChR2 in neurons as Abbott et al. (2009) (Abbott et al. 2009) and Vesuna et al.(2020) (Vesuna et al. 2020), cFos-GFP/mCherry positive neurons within brain slices generated action potentials riding upon depolarization ramp (amplitudes adjusted not to induce action potentials) (*n* = 5 cortical neurons from wt [*n* = 2] or het [*n* = 3] tTA::TetO::*Gabrg2^Q390X^* KI mice) (Supplemental Fig. S1B), while by delivering continuous yellow laser light to activate NpHR in neurons as Gradinaru et al.(2007 and 2008) (Gradinaru et al. 2007, Gradinaru et al. 2008) and Vesuna et al.(2020) (Vesuna et al. 2020), the cFos-GFP/mCherry positive neurons within brain slices exhibited no action potential firings upon depolarization ramp (enough amplitudes adjusted to induce action potentials) (*n* = 6 cortical neurons from the wt [*n* = 3] or het [*n* = 3] cFos-tTA::TetO::*Gabrg2^Q390X^* KI mice) (Supplemental Fig. S1B). Similarly, blue laser ChR2-activation or yellow laser NpHR-activation activated or suppressed VLPO neurons (Supplemental Fig. S1C) (compared to cortical neurons, the VLPO neurons had more depolarized resting membrane potentials [around −50 ~ −58 mV, *n* = 5 neurons from wt *n* = 2 and het *n* = 2 mice]), confirming that blue or yellow laser lights could effectively activate or suppress cortical/subcortical neurons in the brain. In addition, all cFos-GFP positive cells been recorded within the cortex (with pyramidal forms in 40x image Supplemental Fig. S1A) and VLPO (with large dendrites in 40x Supplemental Fig. S1A) showed action potentials with injected currents, which indicates that if cFos-GFP microglia and astrocytes are present, they may represent very small portion of cFos-GFP positive cells in the cortex and VLPO in these transgenic mice. Thus, although some microglia/astrocytes have recently been shown to express cFos in the brain (Aguilar-Delgadillo et al. 2024), their functions in these transgenic mice may not contribute to POA regulated seizures (also see discussion section).

**Fig. 3:**
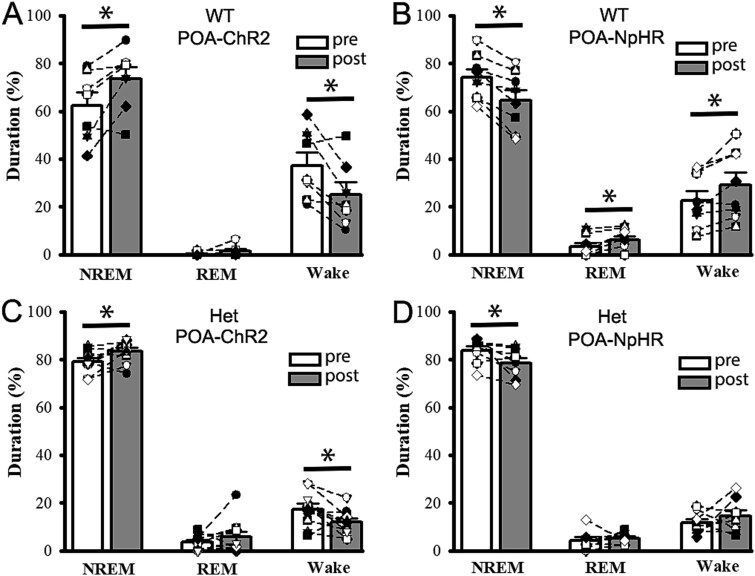
Optogenetic activation/suppression of POA nucleus alone alters NREM/REM sleep and wake periods in cFos-tTA::tetO::Wt and het cFos-tTA::tetO::*Gabrg2^Q390X^* KI mice. Panels A/B show, in the wt mice, the relative changes of NREM/REM sleep and wake period following POA-ChR2 activation (10 min, *n* = 8 mice) or POA-NpHR suppression (10 min, *n* = 7). Panels C/D show, in the het *Gabrg2^Q390X^* KI mice, the relative changes of NREM/REM sleep and wake periods following POA-ChR2 activation (*n* = 10 mice) or POA-NpHR suppression (*n* = 8). *Each data point represents original data from one single mouse* and * significant change with paired t-test *P* < 0.05. Otherwise, paired t-tests are not significant.

Furthermore, with optic cannulas implanted within the POA of the cFos-tTA::TetO::wt and het cFos-tTA::TetO::*Gabrg2^Q390X^* KI mice (shortened as wt and het *Gabrg2^Q390X^* KI mice below), we examined whether optogenetic activation or suppression of the POA nucleus (total 10 min) causally altered brain states in the mice. As we expected, in both the wt and het *Gabrg2^Q390X^* KI mice, after optogenetic POA-ChR2 activation, the mice significantly exhibited more NREM sleep/less wake period (Fig. 3A and C and Table 1). In contrast, after optogenetic POA-NpHR suppression, the wt mice significantly exhibited less NREM and more REM/wake period (Fig. 3B and D and Table 1). Only NREM sleep in the het *Gabrg2^Q390X^* KI mice was significantly decreased by the POA-NpHR suppression. All these optogenetic manipulations will alter cortical neuronal up/down-states during slow-wave oscillations within sleep, which can drive epileptic activity in the het *Gabrg2^Q390X^* KI mice (Zhang et al. 2021).

**Table 1:** Effects of optogenetic manipulation of POA nucleus on sleep and wake pattens.

POA-ChR2 (wt 7, het 10 mice)			
	NREM sleep (%)	REM sleep (%)	Wake (%)
Wt	62.42 ± 5.45% vs 73.53 ± 4.99% (*P =* 0.022)	0.28 ± 0.23% vs 1.39 ± 0.89%	37.30 ± 5.49% vs 25.08 ± 5.25% (*P =* 0.018)
Het	79.12 ± 1.47% vs 83.56 ± 1.48% (*P =* 0.036)	3.5 ± 1.02% vs 5.83 ± 2.23%	17.38 ± 2.33% vs 12.00 ± 1.69% (*P =* 0.042)
POA-NpHR (wt 7, het 10 mice)			
	NREM sleep (%)	REM sleep (%)	Wake (%)
Wt	74.13 ± 3.38% vs 64.65 ± 4.29% (*P =* 0.0006)	3.23 ± 1.55% vs 6.18 ± 1.66% (*P =* 0.027)	22.64 ± 3.98% vs 29.17 ± 5.10% (*P =* 0.012)
Het	83.15 ± 1.87% vs 78.57 ± 2.14% (*P =* 0.043)	4.40 ± 1.41% vs 5.33 ± 0.78%	11.74 ± 1.59% vs 14.58 ± 2.50%

*Each data point represents original data from single mouse.* Except stated, all paired t-tests show no significant difference regarding pre vs postoptogenetic manipulation.

### In het *Gabrg2^Q390X^* KI mice, optogenetic activation of somatosensory cortex alone does not effectively trigger epileptic activity, while optogenetic activation of subcortical POA alone slightly modulates spontaneous epileptic activity in vivo

Our previous work has indicated that sleep-related up-states (in slow-wave oscillations) induced synaptic potentiation in cortical neurons can trigger epileptic activity in het *Gabrg2^Q390X^* KI mice in vivo (Zhang et al. 2021). Thus, we examined whether the optogenetic manipulation of subcortical POA activity could interact with cortical epileptic neurons to generate ictal activity in the het *Gabrg2^Q390X^* KI mice. By using cFos-tTA::TetO::wt and het cFos-tTA::TetO::*Gabrg2^Q390X^* KI mice (shortened as wt and het Gabrg2^Q390X^ KI mice below), we activated epileptic cortical cFos-GFP/mCherry neurons within somatosensory cortex (shortened as S1-ChR2 activation) from mice in a very short period (60 ms, simulating sleep spindles duration in cortical neurons (Kandel and Buzsaki 1997, Bandarabadi et al. 2020, Zhang et al. 2021) without causing kindling) through blue-laser delivery. In the wt mice, we did not find any epileptic activity, while we observed epileptic activity in the het *Gabrg2^Q390X^* KI mice. However, the optogenetic S1-ChR2 activation did not significantly evoke epileptic activity in the het *Gabrg2^Q390X^* KI mice (Supplemental Fig. S2, S1-ChR2: wt [*n* = 7 mice] SWD/PSD/hr pre 7.14 ± 3.71 vs post 5.57 ± 2.69, paired t-test *P* = 0.18; SWD/PSD duration s/hr pre 11.17 ± 5.84 vs post 9.52 ± 4.99, paired t-test *P* = 0.19; het [*n* = 7 mice] SWD/PSD/hr pre 66.14 ± 16.07 vs post 85.14 ± 23.06, paired t-test *P* = 0.08; SWD/PSD duration[s] pre 228.49 ± 87.89 s vs post 307.12 ± 114.78, paired t-test *P* = 0.063). This suggests that without the subcortical POA activation, sleep spindle-like short activation of epileptic cortical neurons within somatosensory cortex is not sufficiently to trigger epileptic activity in the het *Gabrg2^Q390X^* KI mice.

**Fig. 4:**
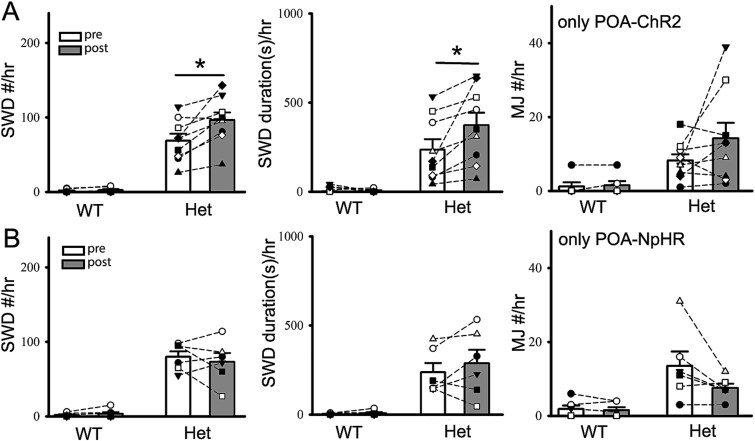
Optogenetic activation of POA nucleus alone slightly modulates epileptic activity in het cFos-tTA::tetO::*Gabrg2^Q390X^* KI mice. Panels show the summary data of relative changes of epileptic SWD/PSD #, SWD/PSD duration and MJ#/hr changes following sole POA-ChR2 activation in mice (panel A, wt 7 and het 9 mice) or POA-NpHR suppression in the mice (panel B, wt 7 and het 6 mice). *Each data point represents original data from one single mouse* and paired t-tests are used with * as significant difference between pre and postoptogenetic manipulation.

Moreover, while optogenetic POA-ChR2 activation was induced in the het cFos-tTA::TetO::*Gabrg2^Q390X^* KI mice, spontaneous epileptic SWD activity (not MJs) (no cortical S1-ChR2 activation) could be slightly enhanced by around 30% (SWD #: het 68.78 ± 9.37 vs 96.33 ± 10.28, 9 mice, paired t-test *P =* 0.004; SWD duration[s]: het 235.96 ± 59.44 vs 373.30 ± 70.18, 9 mice, paired t-test *P =* 0.015, Fig. 4A) (other data values for wt and het included in Table 2). However, optogenetic POA-NpHR suppression did not show much effect on spontaneous epileptic activity (SWDs and MJs) in the wt and het KI mice (data values for wt and het included in Table 2). All these suggest that POA activation alone can influence spontaneous epileptic activity in the het *Gabrg2^Q390X^* KI mice. Due to that the optogenetic POA-ChR2 activation did not synergically coordinate with spontaneous epileptic activity in the mice (as up/down-state alteration within slow-wave oscillation during sleep [Kandel and Buzsaki 1997, Shu et al. 2003]), the optogenetic POA-ChR2 activation generated minor modulatory effects (data values for wt and het included in Table 2) on the spontaneous epileptic activity in the het *Gabrg2^Q390X^* KI mice.

**Table 2:** Effects of optogenetic manipulation of sole POA nucleus on spontaneous epileptic activity in mice.

POA-ChR2 (wt 7, het 9 mice)			
	SWD#/h	SWD duration s/hr	MJ#/hr
Wt	1.29 ± 0.84 vs 2.42 ± 1.34	2.25 ± 1.54 vs 5.04 ± 3.12	1.17 ± 1.17 vs 1.50 ± 1.14
Het	68.78 ± 9.37 vs 96.33 ± 10.28 (*P =* 0.004)	235.96 ± 59.44 vs 373.30 ± 70.18 (*P =* 0.015)	8.22 ± 1.67 vs 14.22 ± 4.21
POA-NpHR (wt 7, het 6 mice)			
	SWD#/hr	SWD duration s/hr	MJ#/hr
Wt	1.85 ± 0.88 vs 3.42 ± 2.09	3.16 ± 1.53 vs 7.84 ± 4.81	1.83 ± 0.98 vs 1.50 ± 0.81
Het	79.83 ± 7.43 vs 73.17 ± 11.82	237.45 ± 51.55 vs 287.38 ± 75.95	13.50 ± 3.92 vs 7.50 ± 1.20

*Each data point represents original data from one single mouse.* Except stated, all paired t-tests show no significant difference regarding pre vs post-optogenetic manipulation.

### Coordinated optogenetic manipulation of the subcortical POA activity and somatosensory epileptic cortical neuron activation regulates seizures in vivo in het *Gabrg2^Q390X^* KI mice

**Fig. 5:**
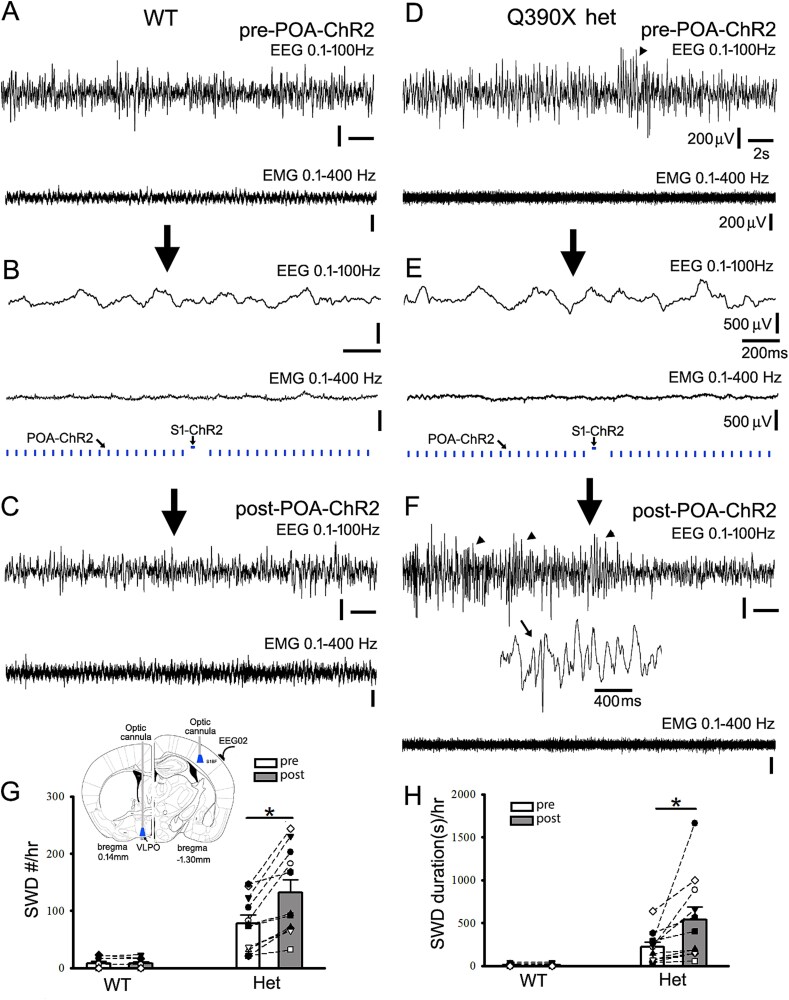
Subcortical POA-ChR2 activation coordinated with short cortical S1-ChR2 activation increases epileptic SWD/PSDs and duration in het cFos-tTA::tetO::*Gabrg2^Q390X^* KI mice. Panels A/C and D/F show simultaneous representative EEG (band filtered 0.1 to 100 Hz) and EMG recordings (band filtered 10 to 400 Hz) from the wt and the het *Gabrg2^Q390X^* KI mice. Panels B/E show the coordinated POA-ChR2 activation and short cortex S1-ChR2 activation for down/up-states within slow-wave oscillations from the wt and het *Gabrg2^Q390X^* KI mice in vivo. In panel F, one SWD event is expanded in small temporal scales under EEG traces. The big arrows between panels show experimental procedure and arrowheads in EEG traces (panels D/F) indicate detected epileptic SWD/PSDs from the het *Gabrg2^Q390X^* KI mice. Scale bars are indicated as labeled. Panels G/H show summary data of epileptic SWD/PSDs and duration changes following POA-ChR2/S1-ChR2. In panel G, one inset shows the experimental design for laser delivery. Wt *n* = 8 mice and het *n* = 11 mice. *Each data point represents original data from one single mouse* and * significant change with paired t-test *P* < 0.05. Otherwise, paired t-tests are not significant.

**Fig. 6:**
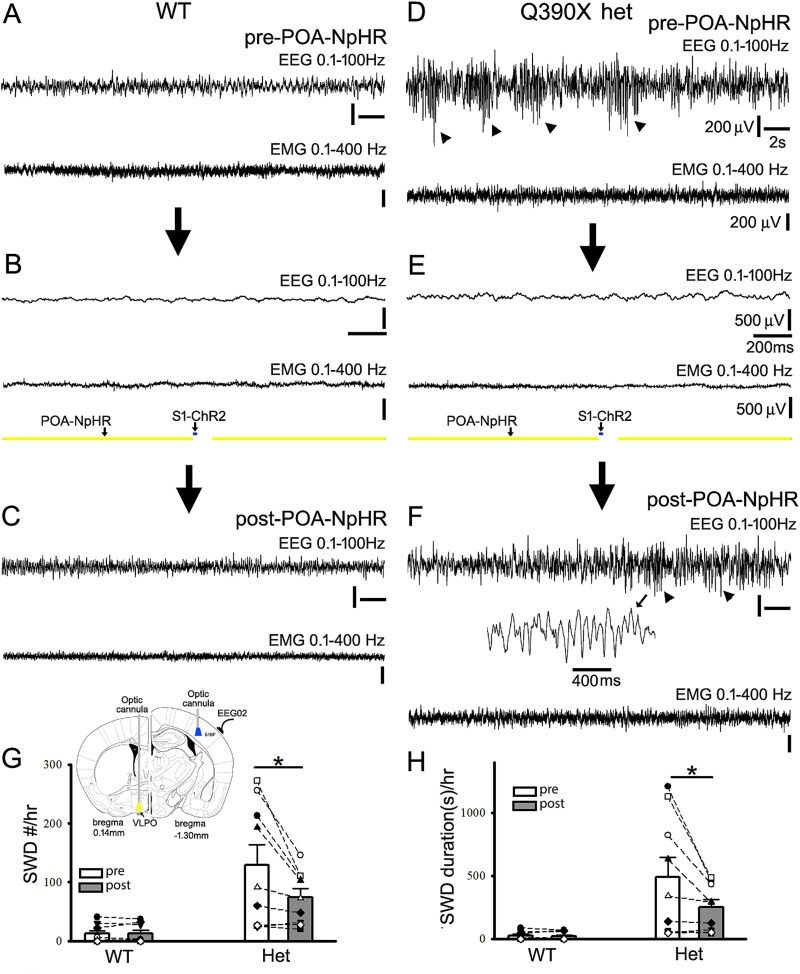
Subcortical POA-NpHR suppression coordinated with short cortical S1-ChR2 activation decreases epileptic SWD/PSDs and duration in het cFos-tTA::tetO::*Gabrg2^Q390X^* KI mice. Panels A/C and D/F show simultaneous representative EEG (band filtered 0.1 to 100 Hz) and EMG recordings (band filtered 10 to 400 Hz) from the wt and the het *Gabrg2^Q390X^* KI mice. Panels B/E show the coordinated POA-NpHR suppression and S1-ChR2 activation in the brain from the wt and het *Gabrg2^Q390X^* KI mice in vivo. In panel F, one SWD event is expanded in small temporal scale from EEG traces. The arrows between panels show experimental procedure and arrowheads in EEG traces (panels D/F) indicate detected epileptic SWD/PSDs from the het *Gabrg2^Q390X^* KI mice. Scale bars are indicated as labeled. Panel G/H shows summary data of epileptic SWD/PSDs and their duration changes following POA-NpHR/S1-ChR2 manipulation. In panel G, one inset shows the experimental design for laser delivery. Wt *n* = 8 mice and het *n* = 9 mice). *Each data point represents original data from one single mouse* and * significant change with paired t-test *P* < 0.05. Otherwise, paired t-tests are not significant.

Neuronal up/down-state alterations during sleep slow-wave oscillations have been suggested to underlie seizure generation in animal models and patients (Halasz et al. 2002, Shu et al. 2003, Beenhakker and Huguenard 2009, Zhang et al. 2021). We reasoned that subcortical POA-activation or suppression should be coordinated with cortical activation of epileptic neuronal engrams/ensembles to produce seizures during sleep. Thus, the optogenetic manipulation of the POA activity was coordinated with short cortical activation by S1-ChR2 in cFos-tTA::TetO::wt and het cFos-tTA::TetO::*Gabrg2^Q390X^* KI mice (shortened as wt and het *Gabrg2^Q390X^* KI mice below) to simulate neuronal up/down state alteration within slow-wave oscillations (0.5 Hz, total 10 minutes, Figs. 5, 6B and E) during sleep (Kandel and Buzsaki 1997, Zhang et al. 2021). As we expected in the wt mice, we found that coordinated POA-ChR2 activation/POA-NpHR suppression with cortical S1-ChR2 activation did not generate any effect on epileptic activity except sleep or wake period alteration, similar to results as Fig. 4 (in wt) (Fig. 5, POA-ChR2/S1-ChR2 wt [*n* = 8 mice]: SWD/PSD/hr pre 8.625 ± 3.34 vs post 7.75 ± 3.13, paired t-test *P* = 0.317; SWD/PSD duration[s] pre 13.28 ± 5.25 vs post 11.28 ± 4.78, paired t-test *P* = 0.053) (Fig. 6, POA-NpHR/S1-ChR2 wt [*n* = 8 mice]: SWD/PSD/hr pre 12.5 ± 5.38 vs post 12.91 ± 5.43, paired t-test *P* = 0.791; SWD/PSD duration[s] pre 25.41 ± 11.34 vs post 20.02 ± 9.49, paired t-test *P* = 0.379). In contrast, after the POA-ChR2 activation was coordinated with a short cortical S1-ChR2 activation in the het *Gabrg2^Q390X^* KI mice, seizures SWD/PSDs were significantly increased with longer duration, compared to prior blue-laser delivery (Fig. 5, het *Gabrg2^Q390X^* KI mice [*n* = 11 mice] POA-ChR2/S1-ChR2: SWD/PSD/hr pre 78.38 ± 14.19 vs post 132.27 ± 22.60, paired t-test *P* = 0.001; SWD/PSD duration[s] pre 221.56 ± 54.25 vs post 538.43 ± 148.09, paired t-test *P* = 0.026). Furthermore, in the het *Gabrg2^Q390X^* KI mice, after the POA-NpHR suppression was coordinated with the short cortical S1-ChR2 activation, epileptic SWD/PSDs were significantly reduced with much shorter duration, compared to prior epileptic events before yellow-laser delivery (Fig. 6 het *Gabrg2^Q390X^* KI mice [*n* = 9] POA-NpHR/S1-ChR2: SWD/PSD/hr pre 129.22 ± 34.61 vs post 74.56 ± 14.98, paired t-test *P* = 0.029; SWD/PSD duration[s] pre 490.96 ± 157.26 vs post 249.89 ± 60.60, paired t-test *P* = 0.043). Moreover, enhanced effects of seizures by the coordinated POA-ChR2/S1-ChR2 manipulation in the het *Gabrg2^Q390X^* KI mice could be reversed by coordinated POA-NpHR/S1-ChR2 manipulation (see Figs 5G and 6G or 5H and 6H), indicating that the POA activity dynamically regulates the cortical epileptic activity in the het *Gabrg2^Q390X^* KI mice and the POA activity needs to coordinate epileptic cortical engrams/ensembles to trigger seizures, compatible with our previous findings and other study.

In addition, sleep spindle generation depends on sleep state and cortical neuron ensemble activation. With the coordinated POA-ChR2/S1-ChR2 optogenetic activation in wt littermate mice in vivo, sleep spindle density was significantly increased (Supplemental Fig. S3 wt, pre 0.0095 ± 0.0010 Hz vs post 0.0170 ± 0.0020 Hz, *n* = 6 mice, paired t-test *P* = 0.015), while in het *Gabrg2^Q390X^* KI mice, sleep spindle density was significantly reduced (Supplemental Fig. S3 het, pre 0.0081 ± 0.0007 Hz vs post 0.0060 ± 0.0005 Hz, *n* = 11 mice, paired t-test *P* = 0.015), indicating that the generation of epileptic activity occludes the generation of sleep spindles in the het *Gabrg2^Q390X^* KI mice.

**Fig. 7:**
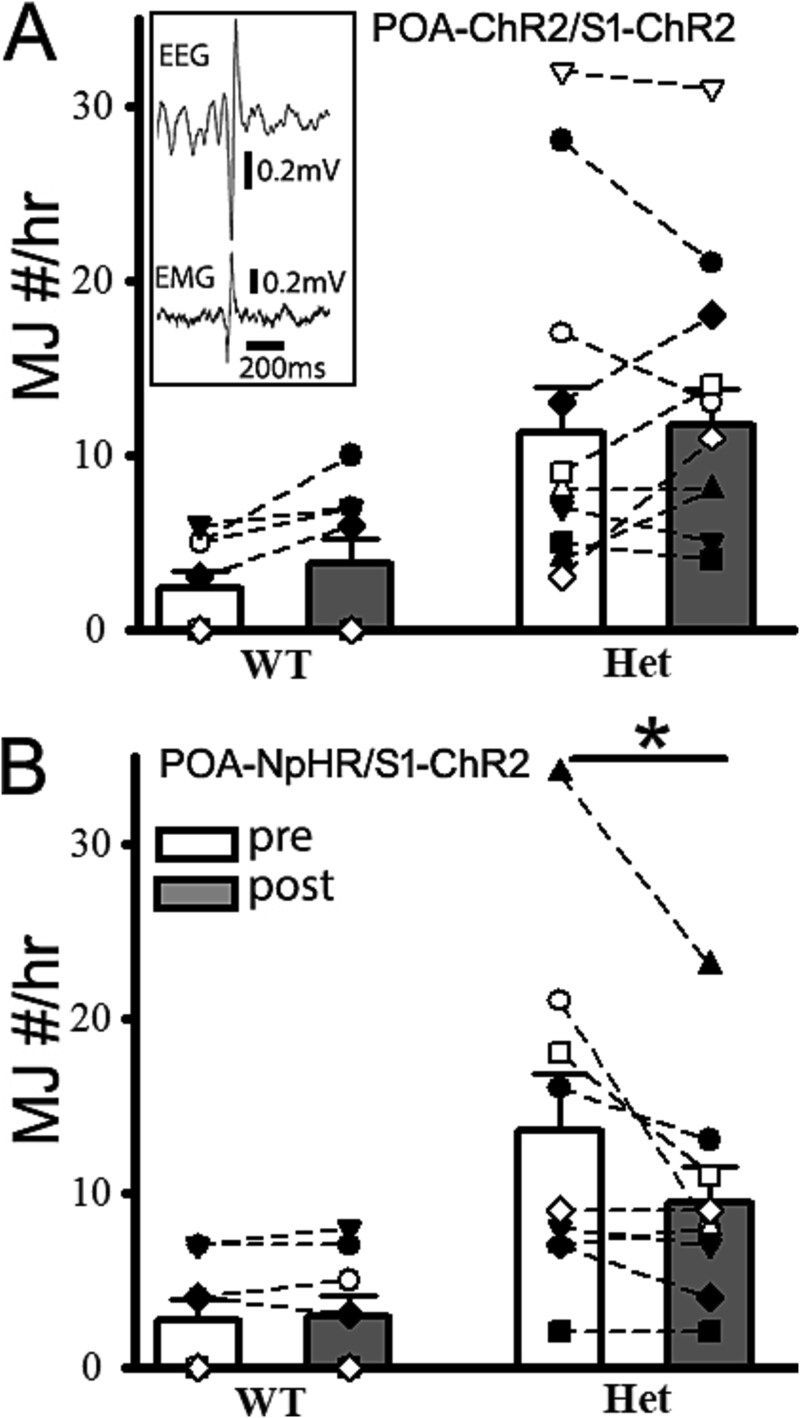
Subcortical POA-NpHR suppression along with short cortical S1-ChR2 activation decreases myoclonic jerks in het cFos-tTA::tetO::*Gabrg2^Q390X^* KI mice. Panel A shows summary MJ changes following POA-ChR2/S1-ChR2 activation. The inset shows the one representative MJ event with EEG/EMG traces. Scale bars are indicated as labeled (wt *n* = 8 mice, het *n* = 10 mice). No significant change with paired t-test. Panel B shows summary MJ changes following POA-NpHR suppression/S1-ChR2 activation. Wt *n* = 8 mice, het *n* = 9 mice. *Each data point represents original data from one single mouse* and * significant change with paired t-test *P* < 0.05. Otherwise, paired t-tests are not significant.

Meanwhile, with coordinated optogenetic activation POA-ChR2/S1-ChR2, we did not observe much effect of myoclonic jerk activity (MJs) (Arain et al. 2015) in the het *Gabrg2^Q390X^* KI mice (Fig. 7, wt, [*n* = 8 mice] POA-ChR2/S1-ChR2: MJs/hr pre 2.37 ± 0.94 vs post 3.75 ± 1.47, paired t-test *P* = 0.073; het [*n* = 10 mice] POA-ChR2/S1-ChR2: MJs/hr pre 11.25 ± 2.69 vs post 11.75 ± 1.99, paired t-test *P* = 0.78). However, with coordinated POA-NpHR suppression with S1-ChR2 activation (POA-NpHR/S1-ChR2), we did observe the reduction of MJs in het *Gabrg2^Q390X^* KI mice (Fig. 8, wt [*n* = 9 mice] POA-NpHR/S1-ChR2: MJs/hr pre 2.75 ± 1.11 vs post 2.88 ± 1.20, paired t-test *P* = 0.59; het [*n* = 10 mice] POA-NpHR/S1-ChR2: MJs/hr pre 13.56 ± 3.27 vs post 9.44 ± 2.02, paired t-test *P* = 0.042), suggesting that myoclonic jerks may have different incident mechanism (Ding and Gallagher 2016, Ding et al. 2019) than SWD/PSDs in the het *Gabrg2^Q390X^* KI mice. However, the subcortical POA may contribute to MJs in this genetic epileptic mouse model, compatible with human clinical dystonia study (Fukuyama et al. 2020, Fotedar and Luders 2024).

## Discussion

In this study we have found that the subcortical POA neurons were active in the brain of the het *Gabrg2^Q390X^* KI mice and the POA activity preceded or/and coincided with epileptic SWD/PSDs in the het *Gabrg2^Q390X^* KI mice. Meanwhile, manipulating subcortical POA activity could alter NREM sleep and wake duration in both wt and het *Gabrg2^Q390X^* KI mice. Most importantly, the short activation of epileptic cortical neurons alone did not effectively trigger seizure activity in the het *Gabrg2^Q390X^* KI mice. In contrast, the coordination of the subcortical POA nucleus activation or suppression with the short activation of epileptic cortical neurons effectively enhanced or suppressed seizures in the het *Gabrg2^Q390X^* KI mice, indicating that the subcortical POA activity can alter the brain state to effectively produce seizures in the het *Gabrg2^Q390X^* KI mice in vivo*,* plus myoclonic jerk activity in the het KI mice*.* Overall, this study discloses an operational mechanism for sleep preferential seizures in this genetic epilepsy model and refractory epilepsy.

In addition, some glial cells have recently been shown to express cFos (Aguilar-Delgadillo et al. 2024), which will drive ChR2/NpHR expression in microglia/astrocytes in cFos::tTA::TetO transgenic mice (Reijmers et al. 2007). However, our morphological data and physiological neuronal firings of cFos-positive neurons (Supplemental Fig. S1) do not support their roles in POA regulating seizures in the het *Gabrg2^Q390X^* KI mice. However, the morphology and physiology experiments do not completely exclude potential glial involvement. Furthermore, optogenetic ChR2-depolarization in microglia/astrocytes (Kir4.1 [Chever et al. 2010, Ohno et al. 2021]) will suppress their intrinsic hyperpolarization activity (Izquierdo et al. 2019), which will decrease their ability to suppress seizures (Badimon et al. 2020, Wu et al. 2020) and hence do not contribute to roles of POA-ChR2/S1-ChR2 activation in enhancing seizures within the het *Gabrg2^Q390X^* KI mice. Furthermore, optogenetic NpHR-activation will hyperpolarize microglia/astrocytes within POA to activate them (Izquierdo et al. 2019), essentially suppressing POA neuronal activity that may contribute to minor effects of POA-NpHR/S1-ChR2 to suppress seizures in the het KI mice. Altogether, cFos expression in potential microglia/astrocytes of this transgenic mice (if this occurs) do not change the main conclusion of subcortical POA activation to provoke seizures during sleep.

Manipulating MnPO nucleus (part of POA, either by direct low-frequency 8 to 13 Hz stimulating or microdialytic infusion of glutamate) has been shown to influence absence seizures (even tonic–clonic seizures by repetitive stimulation kindling in MnPO) in one WAG/Rij rodent model (Suntsova et al. 2009), suggesting that the POA nucleus may involve in seizure generation. However, those manipulation can have effects only when rats are awake or quiet-awake, not sleeping. Moreover, their methods to manipulate MnPO nucleus activate much more neurons other than seizure-related cFos neurons (Suntsova et al. 2009), which makes it difficult to assess the precise mechanism of sleep-active POA neurons on seizures during sleep. Meanwhile, the hypothesis of insufficiency of the preoptic sleep-promoting seizures (Suntsova et al. 2009) cannot explain sleep preferential seizures in genetic epileptic models (Zhang et al. 2021) and patients with refractory epilepsy (St Louis 2011, Romero-Osorio et al. 2018, Rashed et al. 2019, Dell et al. 2021, Grinalds et al. 2023). In our study, only cFos-active neurons from cFos-tTA::TetO mice will be selectively activated by optogenetic methods in both subcortical POA and cortical neurons. Particularly, our data further disclose an operational mechanism in which subcortical POA nucleus activation needs to coordinate with epileptic cortical neuron engrams/ensembles to favorably trigger seizures in het *Gabrg2^Q390X^* KI mice, in the same way by which neuron up/down state alternate during sleep slow-wave oscillations and sleep spindles. We have shown that the short activation of epileptic cortical neurons alone (Supplemental Fig. S2, S1-ChR2 60 ms, simulating sleep spindle duration) cannot effectively trigger epileptic activity in the het *Gabrg2^Q390X^* KI mice, while only subcortical POA-ChR2 activation slightly enhances spontaneous cortical epileptic activity (by 30% increase, while POA-NpHR suppression does not have any effects) (Fig. 4A). Only when subcortical POA-ChR2 activation is coordinated the cortical S1-ChR2 activation (60 ms) can effectively trigger epileptic activity (by almost 80% increase) with much longer duration (by almost 200% increase) in the het *Gabrg2^Q390X^* KI mice (Fig. 5G and H, POA-ChR2/S1-ChR2). In contrast, when the subcortical POA activity is suppressed, the cortical S1-ChR2 activation (60 ms) becomes less effective to trigger epilepsy and remaining epilepsy activity became much shorter in the het *Gabrg2^Q390X^* KI mice (Fig. 6G and H, POA-NpHR/S1-ChR2). These results suggest that *the subcortical POA activation generates particular shortly lived brain states for synchronous cortical neuron activation (engrams) within thalamocortical circuitry* (Contreras and Steriade 1995, Steriade and Contreras 1995, Contreras et al. *1996*, McCormick and Contreras 2001), *which favors either sleep spindle incidence within cortex of the wt mice* (Kandel and Buzsaki 1997, Andrillon et al. 2011, Girardeau and Lopes-Dos-Santos 2021) (Supplemental Fig. S3). Thus, in the het KI mice, POA-induced sleep state will facilitate epileptic cortical engram neuronal synchronization within thalamocortical circuitry (Contreras et al. 1996, Contreras et al. 1997) to dominate brain activity and provoke seizures (Figs. 5 and 6). Consistent with this coordination activation of subcortical POA-ChR2 and cortical S1-ChR2, sleep spindles are increased in wt mice while sleep spindles are decreased in the het *Gabrg2^Q390X^* KI mice due to increased epileptic activity (Supplemental Fig. S3). This indicates that epileptic activity in the het *Gabrg2^Q390X^* KI mice during sleep occludes sleep spindle generation, leading to impaired memory consolidation during sleep in patients with epilepsy (Niethard et al. 2018, Skelin, Zhang et al. 2021, Roebber et al. 2022). This necessity of the coordination between the POA-ChR2 and S1-ChR2 activation is important to regulate epileptic activity in the het *Gabrg2^Q390X^* KI mice and explains why POA-ChR2 activation increased NREM sleep duration and decreased wake duration by a small percentage (around 10% to 20% in Fig. 3A and C), while POA-ChR2/S1-ChR2 activation increased seizures by a larger percentage (around 70% to 89% in Fig. 5G).

Moreover, from translational perspective of treating genetic epilepsy and refractory epilepsy, it is necessary to suppress the sleep preferential seizures in patients with epilepsy, in addition to direct antiepileptic medicine to suppress cortical neuron excitability only (Johannessen and Johannessen 2003, Brandt et al. 2006, White et al. 2007, Sommer et al. 2013). This is indicated by the results of the POA suppression in the het *Gabrg2^Q390X^* KI mice (Fig. 6D–H for POA-NpHR/S1-ChR2), which essentially prevents sleep-related epilepitogenesis in the het *Gabrg2^Q390X^* KI mice (also myoclonic jerks [Fig. 7]). One drug 4-(diethylamino)-benzaldehyde (DEAB) to suppress up-state dependent synaptic potentiation in cortical neurons (Zhang et al. 2021) from the het *Gabrg2^Q390X^* KI mice can be used for this treatment. Compatible with this, DEAB administration (*i.p.*) does attenuate epilepsy (Catron et al. 2023) in the het *Gabrg2^Q390X^* KI model (in its Fig. 5E–H [Catron et al. 2023]).

In addition, the subcortical POA nucleus may contribute to myoclonic jerk generation in het *Gabrg2^Q390X^* KI mice, suggesting that epileptic cortical neuron activation (Contreras and Steriade 1995, Contreras et al. 1997, Kandel and Buzsaki 1997, Huguenard and McCormick 2007) within thalamocortical circuitry interacts with other subcortical circuitry than thalamus to evolve into epileptic MJs in patients with epilepsy. Works on myoclonic seizures in mouse models have indicated that both somatosensory and motor cortex are involved to generate myoclonic jerks (Ding and Gallagher 2016, Ding et al. 2019). Although our cortex S1-ChR2 activation focuses on the somatosensory cortex, with reciprocally connection between the somatosensory and the motor cortex in the brain (Petrof et al. 2015, Suter and Shepherd 2015, Hooks 2017), epileptic activity within somatosensory cortex can influence motor cortex for myoclonic jerk generation (Ding et al. 2019). However, we may need more experiments to support the subcortical POA circuitry involvement of myoclonic jerk generation within the motor cortex.

All together, we show that the subcortical POA (extended VLPO and likely MnPO also) sleep circuitry is active in the het *Gabrg2^Q390X^* KI mice and interacts with epileptic cortical neurons to effectively regulate epileptic activity in the het *Gabrg2^Q390X^* KI mice. This operational mechanism underlies sleep-preferential seizures in genetic epilepsy and refractory epilepsy during sleep. Moreover, it also indicates the necessity to treat sleep disturbance, benefiting patients with refractory epilepsy with better seizure treatment and memory/cognitive recovery.

## Supplementary Material

PreopticArea_Influences_SleepSeizures_062025_supplementalMaterials_bhaf187

Supp_Fig_S1_tTA_tetO_neurons_ChR2_NpHR_021925_bhaf187

Supp_Fig_S2_ChR2_SWDs_111923_bhaf187

Supp_Fig_S3_spindles_bhaf187

Supp_Fig_S4_VLPO_bhaf187

## References

[ref1] Abbott SB, Stornetta RL, Socolovsky CS, West GH, Guyenet PG. 2009. Photostimulation of channelrhodopsin-2 expressing ventrolateral medullary neurons increases sympathetic nerve activity and blood pressure in rats. J Physiol. 587:5613–5631. 10.1113/jphysiol.2009.177535. 19822543 PMC2805374

[ref2] Aguilar-Delgadillo A, Cruz-Mendoza F, Teh S L-d A, Ruvalcaba-Delgadillo Y, Jauregui-Huerta F. 2024. Stress-induced c-fos expression in the medial prefrontal cortex differentially affects the main residing cell phenotypes. Heliyon. 10:e39325. 10.1016/j.heliyon.2024.e39325. 39498004 PMC11532284

[ref3] Ahmed OJ, Vijayan S. 2014. The roles of sleep-wake states and brain rhythms in epileptic seizure onset. J Neurosci. 34:7395–7397. 10.1523/JNEUROSCI.1168-14.2014. 24872545 PMC4035509

[ref4] Alam MA, Kumar S, McGinty D, Alam MN, Szymusiak R. 2014. Neuronal activity in the preoptic hypothalamus during sleep deprivation and recovery sleep. J Neurophysiol. 111:287–299. 10.1152/jn.00504.2013. 24174649 PMC3921380

[ref5] Andrillon T et al. 2011. Sleep spindles in humans: insights from intracranial EEG and unit recordings. J Neurosci. 31:17821–17834. 10.1523/JNEUROSCI.2604-11.2011. 22159098 PMC3270580

[ref6] Arain F, Zhou C, Ding L, Zaidi S, Gallagher MJ. 2015. The developmental evolution of the seizure phenotype and cortical inhibition in mouse models of juvenile myoclonic epilepsy. Neurobiol Dis. 82:164–175. 10.1016/j.nbd.2015.05.016. 26054439 PMC4641014

[ref7] Badimon A et al. 2020. Negative feedback control of neuronal activity by microglia. Nature. 586:417–423. 10.1038/s41586-020-2777-8. 32999463 PMC7577179

[ref8] Bagshaw AP, Rollings DT, Khalsa S, Cavanna AE. 2014. Multimodal neuroimaging investigations of alterations to consciousness: the relationship between absence epilepsy and sleep. Epilepsy Behav. 30:33–37. 10.1016/j.yebeh.2013.09.027. 24139808

[ref9] Bandarabadi M et al. 2020. A role for spindles in the onset of rapid eye movement sleep. Nat Commun. 11:5247. 10.1038/s41467-020-19076-2. 33067436 PMC7567828

[ref10] Barth AL, Gerkin RC, Dean KL. 2004. Alteration of neuronal firing properties after in vivo experience in a FosGFP transgenic mouse. J Neurosci. 24:6466–6475. 10.1523/JNEUROSCI.4737-03.2004. 15269256 PMC6729874

[ref11] Battaglia FP, Sutherland GR, McNaughton BL. 2004. Hippocampal sharp wave bursts coincide with neocortical “up-state” transitions. Learn Mem. 11:697–704. 10.1101/lm.73504. 15576887 PMC534698

[ref12] Beenhakker MP, Huguenard JR. 2009. Neurons that fire together also conspire together: is normal sleep circuitry hijacked to generate epilepsy? Neuron. 62:612–632. 10.1016/j.neuron.2009.05.015. 19524522 PMC2748990

[ref13] Behbehani MM . 1995. Functional characteristics of the midbrain periaqueductal gray. Prog Neurobiol. 46:575–605. 10.1016/0301-0082(95)00009-K. 8545545

[ref14] Beleza P, Pinho J. 2011. Frontal lobe epilepsy. J Clin Neurosci. 18:593–600. 10.1016/j.jocn.2010.08.018. 21349720

[ref15] Bergmann M et al. 2020. A prospective controlled study about sleep disorders in drug resistant epilepsy. Sleep Med. 75:434–440. 10.1016/j.sleep.2020.09.001. 32987342

[ref16] Besing GK, St John EK, Potesta CV, Gallagher MJ, Zhou C. 2022. Artificial sleep-like up/down-states induce synaptic plasticity in cortical neurons from mouse brain slices. Front Cell Neurosci. 16:948327. 10.3389/fncel.2022.948327. 36313618 PMC9615418

[ref17] Bonakis A, Koutroumanidis M. 2009. Epileptic discharges and phasic sleep phenomena in patients with juvenile myoclonic epilepsy. Epilepsia. 50:2434–2445. 10.1111/j.1528-1167.2009.02110.x. 19453715

[ref18] Brandt C, Gastens AM, Sun M, Hausknecht M, Loscher W. 2006. Treatment with valproate after status epilepticus: effect on neuronal damage, epileptogenesis, and behavioral alterations in rats. Neuropharmacology. 51:789–804. 10.1016/j.neuropharm.2006.05.021. 16806297

[ref19] Buchanan GF et al. 2021. Proceedings of the sleep and epilepsy workshop: section 3 mortality: sleep, night, and SUDEP. Epilepsy Curr. 21:15357597211004556. 10.1177/15357597211004556. 33787378 PMC8609595

[ref20] Burnsed J, Skwarzynska D, Wagley PK, Isbell L, Kapur J. 2019. Neuronal circuit activity during neonatal hypoxic-ischemic seizures in mice. Ann Neurol. 86:927–938. 10.1002/ana.25601. 31509619 PMC7025736

[ref21] Caraballo RH, Pasteris MC, Portuondo E, Fortini PS. 2015. Panayiotopoulos syndrome and diffuse paroxysms as the first EEG manifestation at clinical onset: a study of nine patients. Epileptic Disord. 17:143–149. 10.1684/epd.2015.0740. 25895593

[ref22] Cary BA, Turrigiano GG. 2021. Stability of neocortical synapses across sleep and wake states during the critical period in rats. elife. 10:e66304. 10.7554/eLife.66304. PMC8275129 34151775

[ref23] Catron MA et al. 2023. Sleep slow-wave oscillations trigger seizures in a genetic epilepsy model of Dravet syndrome. Brain Commun. 5:fcac332. 10.1093/braincomms/fcac332. 36632186 PMC9830548

[ref24] Chauvette S, Soltani S, Seigneur J, Timofeev I. 2016. In vivo models of cortical acquired epilepsy. J Neurosci Methods. 260:185–201. 10.1016/j.jneumeth.2015.08.030. 26343530 PMC4744568

[ref25] Chever O, Djukic B, McCarthy KD, Amzica F. 2010. Implication of Kir4.1 channel in excess potassium clearance: an in vivo study on anesthetized glial-conditional Kir4.1 knock-out mice. J Neurosci. 30:15769–15777. 10.1523/JNEUROSCI.2078-10.2010. 21106816 PMC6633770

[ref26] Chuhma N, Tanaka KF, Hen R, Rayport S. 2011. Functional connectome of the striatal medium spiny neuron. J Neurosci. 31:1183–1192. 10.1523/JNEUROSCI.3833-10.2011. 21273403 PMC3074638

[ref27] Contreras D, Steriade M. 1995. Cellular basis of EEG slow rhythms: a study of dynamic corticothalamic relationships. J Neurosci. 15:604–622. 10.1523/JNEUROSCI.15-01-00604.1995. 7823167 PMC6578315

[ref28] Contreras D, Destexhe A, Sejnowski TJ, Steriade M. 1996. Control of spatiotemporal coherence of a thalamic oscillation by corticothalamic feedback. Science. 274:771–774. 8864114 10.1126/science.274.5288.771

[ref29] Contreras D, Destexhe A, Steriade M. 1997. Spindle oscillations during cortical spreading depression in naturally sleeping cats. Neuroscience. 77:933–936. 9130774 10.1016/s0306-4522(96)00573-8

[ref30] Dahlberg LS et al. 2018. Responsivity of periaqueductal Gray connectivity is related to headache frequency in episodic migraine. Front Neurol. 9:61. 10.3389/fneur.2018.00061. PMC5816750 29487563

[ref31] Dehghani N et al. 2016. Dynamic balance of excitation and inhibition in human and monkey neocortex. Sci Rep. 6:23176. 10.1038/srep23176. 26980663 PMC4793223

[ref32] Dell KL et al. 2021. Seizure likelihood varies with day-to-day variations in sleep duration in patients with refractory focal epilepsy: a longitudinal electroencephalography investigation. EClinicalMedicine. 37:100934. 10.1016/j.eclinm.2021.100934. 34386736 PMC8343264

[ref33] Ding L, Gallagher MJ. 2016. Dynamics of sensorimotor cortex activation during absence and myoclonic seizures in a mouse model of juvenile myoclonic epilepsy. Epilepsia. 57:1568–1580. 10.1111/epi.13493. 27573707 PMC5056152

[ref34] Ding L, Satish S, Zhou C, Gallagher MJ. 2019. Cortical activation in generalized seizures. Epilepsia. 60:1932–1941. 10.1111/epi.16306. 31368118 PMC6732052

[ref35] Fotedar N, Luders HO. 2024. Nocturnal paroxysmal dystonia to sleep-related hypermotor epilepsy: a critical review. Epilepsia. 65:2506–2518. 10.1111/epi.18067. 39046177

[ref36] Fukuyama K, Fukuzawa M, Shiroyama T, Okada M. 2020. Pathomechanism of nocturnal paroxysmal dystonia in autosomal dominant sleep-related hypermotor epilepsy with S284L-mutant alpha4 subunit of nicotinic ACh receptor. Biomed Pharmacother. 126:110070. 10.1016/j.biopha.2020.110070. 32169758

[ref37] Garcia-Cabrero AM et al. 2012. Laforin and Malin deletions in mice produce similar neurologic impairments. J Neuropathol Exp Neurol. 71:413–421. 10.1097/NEN.0b013e318253350f. 22487859

[ref38] Gibbon FM, Maccormac E, Gringras P. 2019. Sleep and epilepsy: unfortunate bedfellows. Arch Dis Child. 104:189–192. 10.1136/archdischild-2017-313421. 30266875 PMC6362435

[ref39] Girardeau G, Lopes-Dos-Santos V. 2021. Brain neural patterns and the memory function of sleep. Science. 374:560–564. 10.1126/science.abi8370. 34709916 PMC7611961

[ref40] Gradinaru V et al. 2007. Targeting and readout strategies for fast optical neural control in vitro and in vivo. J Neurosci. 27:14231–14238. 10.1523/JNEUROSCI.3578-07.2007. 18160630 PMC6673457

[ref41] Gradinaru V, Thompson KR, Deisseroth K. 2008. eNpHR: a Natronomonas halorhodopsin enhanced for optogenetic applications. Brain Cell Biol. 36:129–139. 10.1007/s11068-008-9027-6. 18677566 PMC2588488

[ref42] Grinalds MS, Yoder C, Krauss Z, Chen AM, Rhoney DH. 2023. Scoping review of rational polytherapy in patients with drug-resistant epilepsy. Pharmacotherapy. 43:53–84. 10.1002/phar.2748. 36484111 PMC10107532

[ref43] Gvilia I et al. 2017. The role of adenosine in the maturation of sleep homeostasis in rats. J Neurophysiol. 117:327–335. 10.1152/jn.00675.2016. 27784808 PMC5225952

[ref44] Halasz P, Terzano MG, Parrino L. 2002. Spike-wave discharge and the microstructure of sleep-wake continuum in idiopathic generalised epilepsy. Neurophysiologie Clinique-Clinical Neurophysiology. 32:38–53. 10.1016/S0987-7053(01)00290-8. 11915485

[ref45] Halasz P, Kelemen A, Szucs A. 2013. The role of NREM sleep micro-arousals in absence epilepsy and in nocturnal frontal lobe epilepsy. Epilepsy Res. 107:9–19. 10.1016/j.eplepsyres.2013.06.021. 24050971

[ref46] Hooks BM . 2017. Sensorimotor convergence in circuitry of the motor cortex. Neuroscientist. 23:251–263. 10.1177/1073858416645088. 27091827

[ref47] Huguenard JR, McCormick DA. 2007. Thalamic synchrony and dynamic regulation of global forebrain oscillations. Trends Neurosci. 30:350–356. 10.1016/j.tins.2007.05.007. 17544519

[ref48] Izquierdo P, Attwell D, Madry C. 2019. Ion channels and receptors as determinants of microglial function. Trends Neurosci. 42:278–292. 10.1016/j.tins.2018.12.007. 30678990

[ref49] Johannessen CU, Johannessen SI. 2003. Valproate: past, present, and future. CNS Drug Rev. 9:199–216. 10.1111/j.1527-3458.2003.tb00249.x. 12847559 PMC6741662

[ref50] Kandel A, Buzsaki G. 1997. Cellular-synaptic generation of sleep spindles, spike-and-wave discharges, and evoked thalamocortical responses in the neocortex of the rat. J Neurosci. 17:6783–6797. 10.1523/JNEUROSCI.17-17-06783.1997. 9254689 PMC6573130

[ref51] Kang JQ, Shen W, Zhou C, Xu D, Macdonald RL. 2015. The human epilepsy mutation GABRG2(Q390X) causes chronic subunit accumulation and neurodegeneration. Nat Neurosci. 18:988–996. 10.1038/nn.4024. 26005849 PMC4482801

[ref52] Konduru SS et al. 2021. Sleep deprivation exacerbates seizures and diminishes GABAergic tonic inhibition. Ann Neurol. 90:840–844. 10.1002/ana.26208. 34476841 PMC8530964

[ref53] Levenstein D, Watson BO, Rinzel J, Buzsaki G. 2017. Sleep regulation of the distribution of cortical firing rates. Curr Opin Neurobiol. 44:34–42. 10.1016/j.conb.2017.02.013. 28288386 PMC5511069

[ref54] Levesque M, Macey-Dare ADB, Wang S, Avoli M. 2021. Evolution of interictal spiking during the latent period in a mouse model of mesial temporal lobe epilepsy. Curr Res Neurobiol. 2:100008. 10.1016/j.crneur.2021.100008. 36246508 PMC9559106

[ref55] Levesque M, Wang S, Macey-Dare ADB, Salami P, Avoli M. 2023. Evolution of interictal activity in models of mesial temporal lobe epilepsy. Neurobiol Dis. 180:106065. 10.1016/j.nbd.2023.106065. 36907521

[ref56] Liang X et al. 2024. Dysregulation of the suprachiasmatic nucleus disturbs the circadian rhythm and aggravates epileptic seizures by inducing hippocampal GABAergic dysfunction in C57BL/6 mice. J Pineal Res. 76:e12993. 10.1111/jpi.12993. 39054842

[ref57] Liguori C et al. 2021. Sleep disorders and late-onset epilepsy of unknown origin: understanding new trajectories to brain amyloidopathy. Mech Ageing Dev. 194:111434. 10.1016/j.mad.2021.111434. 33444630

[ref58] Lim AS et al. 2014. Sleep is related to neuron numbers in the ventrolateral preoptic/intermediate nucleus in older adults with and without Alzheimer’s disease. Brain. 137:2847–2861. 10.1093/brain/awu222. 25142380 PMC4163039

[ref59] Lu J, Greco MA, Shiromani P, Saper CB. 2000. Effect of lesions of the ventrolateral preoptic nucleus on NREM and REM sleep. J Neurosci. 20:3830–3842. 10.1523/JNEUROSCI.20-10-03830.2000. 10804223 PMC6772663

[ref60] Manokaran RK, Tripathi M, Chakrabarty B, Pandey RM, Gulati S. 2020. Sleep abnormalities and polysomnographic profile in children with drug-resistant epilepsy. Seizure. 82:59–64. 10.1016/j.seizure.2020.09.016. 33011589

[ref61] Marciszewski KK et al. 2018. Changes in brainstem pain modulation circuitry function over the migraine cycle. J Neurosci. 38:10479–10488. 10.1523/JNEUROSCI.1088-18.2018. 30341182 PMC6596255

[ref62] Marciszewski KK et al. 2019. Fluctuating regional brainstem diffusion imaging measures of microstructure across the migraine cycle. eNeuro. 6:ENEURO.0005–ENEU19.2019. 10.1523/ENEURO.0005-19.2019. 31300542 PMC6658917

[ref63] McCormick DA, Contreras D. 2001. On the cellular and network bases of epileptic seizures. Annu Rev Physiol. 63:815–846. 10.1146/annurev.physiol.63.1.815. 11181977

[ref64] Mohns EJ, Karlsson KA, Blumberg MS. 2006. The preoptic hypothalamus and basal forebrain play opposing roles in the descending modulation of sleep and wakefulness in infant rats. Eur J Neurosci. 23:1301–1310. 10.1111/j.1460-9568.2006.04652.x. 16553791 PMC2645537

[ref65] Ng M, Pavlova M. 2013. Why are seizures rare in rapid eye movement sleep? Review of the frequency of seizures in different sleep stages. Epilepsy Res Treat. 2013:932790. 23853720 10.1155/2013/932790 PMC3703322

[ref66] Ngo HV, Fell J, Staresina B. 2020. Sleep spindles mediate hippocampal-neocortical coupling during long-duration ripples. elife. 9:e57011. 10.7554/eLife.57011. PMC7363445 32657268

[ref67] Niethard N, Ngo HV, Ehrlich I, Born J. 2018. Cortical circuit activity underlying sleep slow oscillations and spindles. Proc Natl Acad Sci USA. 115:E9220–E9229. 10.1073/pnas.1805517115. 30209214 PMC6166829

[ref68] Ohno Y, Kunisawa N, Shimizu S. 2021. Emerging roles of astrocyte Kir4.1 channels in the pathogenesis and treatment of brain diseases. Int J Mol Sci. 22:10236. 10.3390/ijms221910236. PMC8508600 34638578

[ref69] Papale LA et al. 2013. Altered sleep regulation in a mouse model of SCN1A-derived genetic epilepsy with febrile seizures plus (GEFS+). Epilepsia. 54:625–634. 10.1111/epi.12060. 23311867 PMC3703918

[ref70] Pavlova MK et al. 2021. Proceedings of the sleep and epilepsy workgroup: section 2 comorbidities: sleep related comorbidities of epilepsy. Epilepsy Curr 15357597211004549. 21:210–214. 10.1177/15357597211004549. PMC8609600 33843327

[ref71] Petrof I, Viaene AN, Sherman SM. 2015. Properties of the primary somatosensory cortex projection to the primary motor cortex in the mouse. J Neurophysiol. 113:2400–2407. 10.1152/jn.00949.2014. 25632081 PMC4416606

[ref72] Rashed HR et al. 2019. Refractory epilepsy and obstructive sleep apnea: is there an association? Egyptian Journal of Neurology Psychiatry and Neurosurgery. 55. 10.1186/s41983-019-0072-0.

[ref73] Reijmers LG, Perkins BL, Matsuo N, Mayford M. 2007. Localization of a stable neural correlate of associative memory. Science. 317:1230–1233. 10.1126/science.1143839. 17761885

[ref74] Roebber JK, Lewis PA, Crunelli V, Navarrete M, Hamandi K. 2022. Effects of anti-seizure medication on sleep spindles and slow waves in drug-resistant epilepsy. Brain Sci. 12:1288. 10.3390/brainsci12101288. PMC9599317 36291222

[ref75] Romero-Osorio O, Gil-Tamayo S, Narino D, Rosselli D. 2018. Changes in sleep patterns after vagus nerve stimulation, deep brain stimulation or epilepsy surgery: systematic review of the literature. Seizure-European Journal of Epilepsy. 56:4–8. 10.1016/j.seizure.2018.01.022. 29414594

[ref76] Roy DS et al. 2022. Brain-wide mapping reveals that engrams for a single memory are distributed across multiple brain regions. Nat Commun. 13:1799. 10.1038/s41467-022-29384-4. 35379803 PMC8980018

[ref77] Saper CB, Chou TC, Scammell TE. 2001. The sleep switch: hypothalamic control of sleep and wakefulness. Trends Neurosci. 24:726–731. 10.1016/S0166-2236(00)02002-6. 11718878

[ref78] Saper CB, Scammell TE, Lu J. 2005. Hypothalamic regulation of sleep and circadian rhythms. Nature. 437:1257–1263. 10.1038/nature04284. 16251950

[ref79] Saper CB, Fuller PM, Pedersen NP, Lu J, Scammell TE. 2010. Sleep state switching. Neuron. 68:1023–1042. 10.1016/j.neuron.2010.11.032. 21172606 PMC3026325

[ref80] Scammell TE, Arrigoni E, Lipton JO. 2017. Neural circuitry of wakefulness and sleep. Neuron. 93:747–765. 10.1016/j.neuron.2017.01.014. 28231463 PMC5325713

[ref81] Schoonjans AS, De Keersmaecker S, Van Bouwel M, Ceulemans B. 2019. More daytime sleepiness and worse quality of sleep in patients with Dravet syndrome compared to other epilepsy patients. Eur J Paediatr Neurol. 23:61–69. 10.1016/j.ejpn.2018.09.012. 30340858

[ref82] Shu Y, Hasenstaub A, McCormick DA. 2003. Turning on and off recurrent balanced cortical activity. Nature. 423:288–293. 10.1038/nature01616. 12748642

[ref83] Silva C, McNaughton N. 2019. Are periaqueductal gray and dorsal raphe the foundation of appetitive and aversive control? A comprehensive review. Prog Neurobiol. 177:33–72. 10.1016/j.pneurobio.2019.02.001. 30786258

[ref84] Skelin I et al. 2021. Coupling between slow waves and sharp-wave ripples engages distributed neural activity during sleep in humans. Proc Natl Acad Sci USA. 118:e2012075118. 10.1073/pnas.2012075118. PMC8166184 34001599

[ref85] Sommer BR, Mitchell EL, Wroolie TE. 2013. Topiramate: effects on cognition in patients with epilepsy, migraine headache and obesity. Ther Adv Neurol Disord. 6:211–227. 10.1177/1756285613481257. 23858325 PMC3707352

[ref86] St Louis EK . 2011. Sleep and epilepsy: strange bedfellows No more. Minerva Pneumol. 50:159–176. 23539488 PMC3608109

[ref87] Steel D, Symonds JD, Zuberi SM, Brunklaus A. 2017. Dravet syndrome and its mimics: beyond SCN1A. Epilepsia. 58:1807–1816. 10.1111/epi.13889. 28880996

[ref88] Steriade M, Contreras D. 1995. Relations between cortical and thalamic cellular events during transition from sleep patterns to paroxysmal activity. J Neurosci. 15:623–642. 10.1523/JNEUROSCI.15-01-00623.1995. 7823168 PMC6578306

[ref89] Suntsova N, Szymusiak R, Alam MN, Guzman-Marin R, McGinty D. 2002. Sleep-waking discharge patterns of median preoptic nucleus neurons in rats. J Physiol. 543:665–677. 10.1113/jphysiol.2002.023085. 12205198 PMC2290500

[ref90] Suntsova N et al. 2009. A role for the preoptic sleep-promoting system in absence epilepsy. Neurobiol Dis. 36:126–141. 10.1016/j.nbd.2009.07.005. 19631751 PMC2757065

[ref91] Suter BA, Shepherd GM. 2015. Reciprocal interareal connections to corticospinal neurons in mouse M1 and S2. J Neurosci. 35:2959–2974. 10.1523/JNEUROSCI.4287-14.2015. 25698734 PMC4331623

[ref92] Timofeev I, Steriade M. 1998. Cellular mechanisms underlying intrathalamic augmenting responses of reticular and relay neurons. J Neurophysiol. 79:2716–2729. 10.1152/jn.1998.79.5.2716. 9582240

[ref93] Torao-Angosto M, Manasanch A, Mattia M, Sanchez-Vives MV. 2021. Up and down states during slow oscillations in slow-wave sleep and different levels of Anesthesia. Front Syst Neurosci. 15:609645. 10.3389/fnsys.2021.609645. 33633546 PMC7900541

[ref94] Tukker JJ, Beed P, Schmitz D, Larkum ME, Sachdev RNS. 2020. Up and down states and memory consolidation across somatosensory, entorhinal, and hippocampal cortices. Front Syst Neurosci. 14:22. 10.3389/fnsys.2020.00022. 32457582 PMC7227438

[ref95] Van Nuland A et al. 2021. Sleep in Dravet syndrome: a parent-driven survey. Seizure. 85:102–110. 10.1016/j.seizure.2020.12.021. 33453590

[ref96] Verbeek NE et al. 2015. Seizure precipitants in Dravet syndrome: what events and activities are specifically provocative compared with other epilepsies? Epilepsy Behav. 47:39–44. 10.1016/j.yebeh.2015.05.008. 26021464

[ref97] Vesuna S et al. 2020. Deep posteromedial cortical rhythm in dissociation. Nature. 586:87–94. 10.1038/s41586-020-2731-9. 32939091 PMC7553818

[ref98] Warner TA, Liu Z, Macdonald RL, Kang J-Q. 2017. Heat induced temperature dysregulation and seizures in Dravet syndrome/GEFS+ Gabrg2+/Q390X mice. Epilepsy Res. 134:1–8. 10.1016/j.eplepsyres.2017.04.020. 28505490 PMC5512282

[ref99] Weber F et al. 2018. Regulation of REM and non-REM sleep by periaqueductal GABAergic neurons. Nat Commun. 9:354. 10.1038/s41467-017-02765-w. 29367602 PMC5783937

[ref100] White HS, Smith MD and Wilcox KS. 2007. Mechanisms of action of antiepileptic drugs. Int Rev Neurobiol 81: 85–110. 10.1016/S0074-7742(06)81006-8. 17433919

[ref101] Wright KM, McDannald MA. 2019. Ventrolateral periaqueductal gray neurons prioritize threat probability over fear output. elife. 8:e45013. 10.7554/eLife.45013. PMC6435320 30843787

[ref102] Wu W et al. 2020. Microglial depletion aggravates the severity of acute and chronic seizures in mice. Brain Behav Immun. 89:245–255. 10.1016/j.bbi.2020.06.028. 32621847 PMC7572576

[ref103] Yu FH et al. 2006. Reduced sodium current in GABAergic interneurons in a mouse model of severe myoclonic epilepsy in infancy. Nat Neurosci. 9:1142–1149. 10.1038/nn1754. 16921370

[ref104] Zhang CQ et al. 2021. Impaired state-dependent potentiation of GABAergic synaptic currents triggers seizures in a genetic generalized epilepsy model. Cereb Cortex. 31:768–784. 10.1093/cercor/bhaa256. 32930324 PMC7906787

[ref105] Zhong P et al. 2019. Control of non-REM sleep by midbrain Neurotensinergic neurons. Neuron. 104:795–809.e6. 10.1016/j.neuron.2019.08.026. 31582313

