# Correction to: Preoptic area influences sleep-related seizures in a genetic epilepsy mouse model

**DOI:** 10.1093/cercor/bhag005

**Published:** 2026-01-23

This is a correction to: Cobie Victoria Potesta, Madeleine Sandra Cargile, Andrea Yan, Sarah Xiong, Robert L Macdonald, Martin J Gallagher, Chengwen Zhou, Preoptic area influences sleep-related seizures in a genetic epilepsy mouse model, *Cerebral Cortex*, Volume 35, Issue 7, July 2025, 10.1093/cercor/bhaf187

The originally published version of the manuscript lacked Figure S1, S2, S3 and S4 in the Supplementary data section, having only the legends to the images. Correction has been made to the article and the images for the respective figures added.

