# Supplementary material for: Preoptic area influences sleep-related seizures in a genetic epilepsy mouse model
Source: Cereb Cortex. 2025 Jul 22;35(7):bhaf187. doi: 10.1093/cercor/bhaf187 (PMC12281508; doi:10.1093/cercor/bhaf187)
Supplement: PreopticArea_Influences_SleepSeizures_062025_supplementalMaterials_bhaf187 [file preopticarea_influences_sleepseizures_062025_supplementalmaterials_bhaf187.docx]

Preoptic area influences sleep-related seizures in a genetic epilepsy mouse model

Running title: Preoptic area influences sleep-related seizures

Cobie Victoria Potesta^1*^, Madeleine Sandra Cargile^2*^, Andrea Yan^&^, Sarah Xiong^&^, Robert L. Macdonald^1^, Martin J. Gallagher^1,3^, Chengwen Zhou^1,3†^

Department of Neurology^1^, Vanderbilt Brain Institute and Neuroscience graduate program^3^, Vanderbilt University Medical Center, Nashville, TN 37232

Speech, Language and Hearing Science Program, Auburn University^2^, Auburn, AL 36849

^†^ To whom correspondence should be addressed:

Chengwen Zhou, Ph.D., Vanderbilt University Medical Center Dept. Neurology

Medical Research Building III, Room 6136, 465 21st Ave South, Nashville, TN 37232-8552

Tel: 615-322-5983 and E-mail: [chengwen.zhou@vanderbilt.edu](mailto:chengwen.zhou@vanderbilt.edu) and [chengwen.zhou@vumc.org](mailto:chengwen.zhou@vumc.org)

* equally contribute to the work

^&^ Andrea Yan (from Carroll senior high school, Southlake, TX 76092) and Sarah Xiong (from Adlai E. Stevenson High school, Lincolnshire, IL 60069) were summer students.

**Supplemental Figure S1. cFos-GFP positive neurons in somatosensory cortical and POA neurons from cFos-tTA::tetO::wt and het cFos-tTA::tetO::*Gabrg2^Q390X^* KI mice**. Panel A shows cFos-GFP positive neurons (40X GFP) within cortex and VLPO nucleus from wt and the het *Gabrg2^Q390X^* KI mice with more GFP-positive neurons in the het KI mice. Panel B (top) shows that ChR2-activation by 20 Hz blue laser (473nm, 1 ms duration) evokes action potentials riding on the depolarization ramp [n=5 cortical neurons from wt (n=2) or het (n=3) mice]. Panel B (lower) shows that NpHR-activation by continuous yellow laser (590nm) completely suppresses the action potentials generated by depolarization pulse (n=6 cortical neurons from wt n=3 or het n=3 mice). Panel C shows similar effects of optogenetic manipulation on VLPO neuronal firings (n=5 neurons from wt or het n=2 mice each). Neuronal resting membrane potentials of neurons and scale bars are indicated as labeled.

**Supplemental Figure S2. Short cortex S1-ChR2 activation alone does not effectively trigger epileptic activity *in vivo* in het cFos-tTA::tetO::*Gabrg2^Q390X^* KI mice**. Panels A/B show summary data of epileptic SWD/PSDs and their duration changes following sole cortex S1-ChR2 activation. The inset in panel A shows the one representative experimental design for laser delivery. Wt n=7 mice, het n=7 mice. *Each data point represents original data from one single mouse* and no significant changes with paired t-test.

**Supplemental Figure S3. Coordinated POA-ChR2 activation with short cortex S1-ChR2 activation does not increase sleep spindles in het cFos-tTA::tetO::*Gabrg2^Q390X^* KI mice**. Panels A/B shows summary data of sleep spindle density following POA-ChR2/S1-ChR2 activation in wt and het KI mice. wt n=6 mice, het n=11 mice. *Each data point represents original data from one single mouse* and * significant change with paired t-test p<0.05. Otherwise, paired t-tests are not significant.

**Supplemental Figure S4. Optic cannula tracks within the brain.** Panels show optic cannula tracks within brain sections(50µm thickness). The tissue holes along cannula track are marked with black arrows. The VLPO nucleus is labeled within lower brain sections with anatomical landmarks (3V as 3^rd^ ventricle, ACA as anterior commissure ant and ACP as anterior commissure posterior).
