# Supplementary figures and images for: Preoptic area influences sleep-related seizures in a genetic epilepsy mouse model
Source: Cereb Cortex. 2025 Jul 22;35(7):bhaf187. doi: 10.1093/cercor/bhaf187 (PMC12281508; doi:10.1093/cercor/bhaf187)

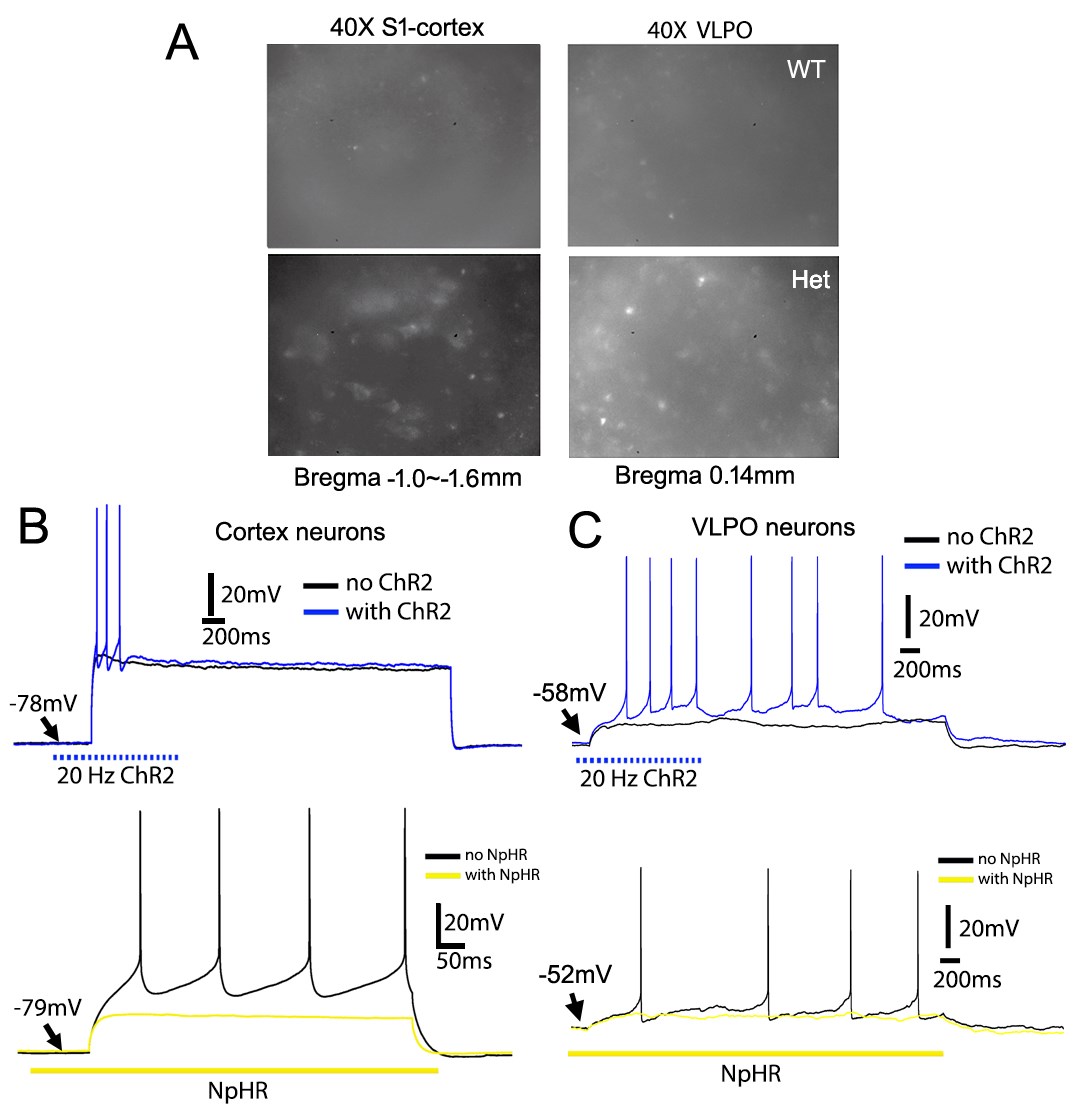

Supplement: Supp_Fig_S1_tTA_tetO_neurons_ChR2_NpHR_021925_bhaf187 [file supp_fig_s1_tta_teto_neurons_chr2_nphr_021925_bhaf187.jpeg]

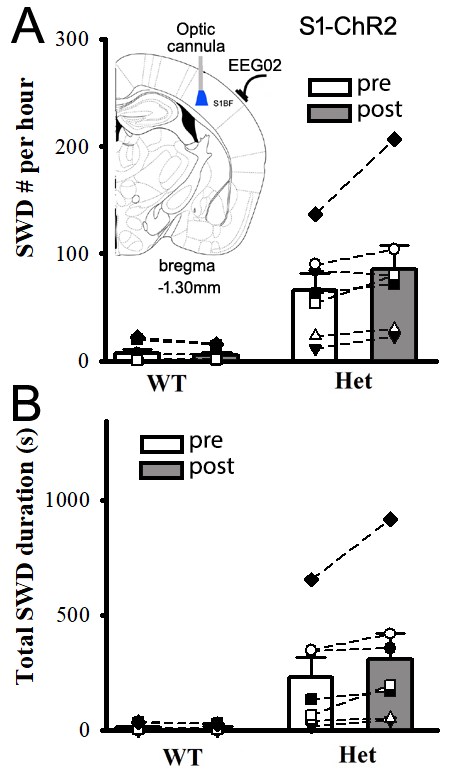

Supplement: Supp_Fig_S2_ChR2_SWDs_111923_bhaf187 [file supp_fig_s2_chr2_swds_111923_bhaf187.jpeg]

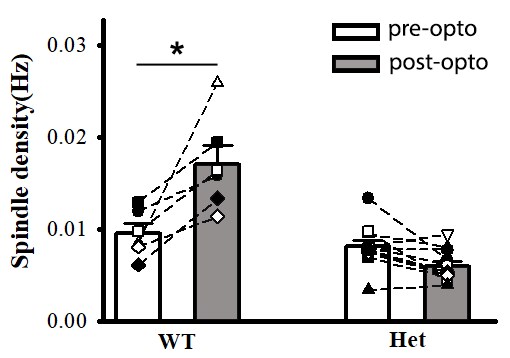

Supplement: Supp_Fig_S3_spindles_bhaf187 [file supp_fig_s3_spindles_bhaf187.jpeg]

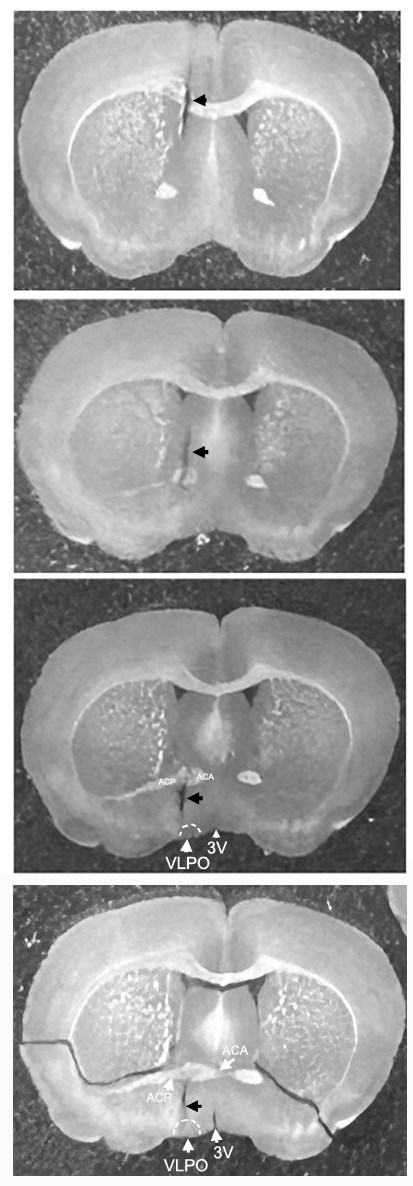

Supplement: Supp_Fig_S4_VLPO_bhaf187 [file supp_fig_s4_vlpo_bhaf187.jpeg]
